# Visible‐Light‐Driven Hydrogen Evolution Using Planarized Conjugated Polymer Photocatalysts

**DOI:** 10.1002/ange.201510542

**Published:** 2015-12-22

**Authors:** Reiner Sebastian Sprick, Baltasar Bonillo, Rob Clowes, Pierre Guiglion, Nick J. Brownbill, Benjamin J. Slater, Frédéric Blanc, Martijn A. Zwijnenburg, Dave J. Adams, Andrew I. Cooper

**Affiliations:** ^1^ Department of Chemistry and Centre for Materials DiscoveryUniversity of LiverpoolCrown StreetLiverpoolL69 7ZDUK; ^2^ Department of ChemistryUniversity College London20 Gordon StreetLondonWC1H 0AJUK; ^3^ Stephenson Institute for Renewable EnergyUniversity of LiverpoolCrown StreetLiverpoolL69 7ZDUK

**Keywords:** Konjugierte Polymere, Photokatalyse, Planarisierung, Wasserspaltung

## Abstract

Linear poly(p‐phenylene)s are modestly active UV photocatalysts for hydrogen production in the presence of a sacrificial electron donor. Introduction of planarized fluorene, carbazole, dibenzo[b,d]thiophene or dibenzo[b,d]thiophene sulfone units greatly enhances the H_2_ evolution rate. The most active dibenzo[b,d]thiophene sulfone co‐polymer has a UV photocatalytic activity that rivals TiO_2_, but is much more active under visible light. The dibenzo[b,d]thiophene sulfone co‐polymer has an apparent quantum yield of 2.3 % at 420 nm, as compared to 0.1 % for platinized commercial pristine carbon nitride.

## Graphical abstract

**Großer Schub für die H_2_‐Produktion**: Die Einführung von planarisierten Fluoren‐, Carbazol‐, Dibenzo[*b,d*]thiophen‐ oder Dibenzo[*b,d*]thiophensulfon‐Einheiten in lineare Poly(*p*‐phenylene) führt zu einer drastisch erhöhten Aktivität in der durch Licht vermittelten Wasserstoffentwicklung. Das aktivste Copolymer, dasjenige mit Dibenzo[*b,d*]thiophensulfon, erreicht die UV‐Aktivität von TiO_2_, ist aber deutlich aktiver unter sichtbarem Licht.

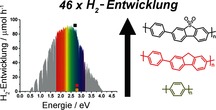

There has been a surge of interest recently in light‐driven water splitting using organic rather than inorganic photocatalysts. The most widely investigated organic material is graphitic carbon nitride (g‐C_3_N_4_).^1^, ^2^, ^3^, ^4^, ^5^, ^6^ A few other nitrogen‐containing organic polymers were also studied, such as doped poly(triazine imide)s,^7^ heptazine networks,^8^ poly(azomethine)s,^9^ hydrazone‐based covalent organic frameworks,^10^ triazine‐based frameworks,^11^, ^12^ triphenyl‐azine frameworks,^13^ and an organic push–pull polymer network/rutile composite.^14^ We recently showed that conjugated microporous polymers (CMPs) also have potential for photocatalytic hydrogen evolution, and in particular that their synthesis lends itself to fine control over photophysical properties, such as the optical gap.^15^, ^16^

Linear conjugated organic polymers are relatively unexplored for light‐driven water splitting, despite the wealth of knowledge that exists in terms of solar energy harvesting in organic photovoltaics. Poly(*p*‐phenylene) loaded with ruthenium was reported to have modest photocatalytic activity under >290 nm irradiation, and low activity when visible irradiation (>400 nm) was used.^17^ Poly(3‐hexylthiophene) (P3HT) was used in a composite with g‐C_3_N_4_,^18^ but platinum‐loaded P3HT by itself showed very little hydrogen evolution under >400 nm irradiation.^17^, ^18^ Polyaniline was also studied in a composite with CdS,^19^ which is an active (but unstable) inorganic catalyst for visible light‐driven hydrogen evolution.

To achieve high water splitting activity, a photocatalyst must thermodynamically drive the proton reduction and water oxidation half reactions. Also, photoexcited state lifetimes and charge‐carrier mobilities in the catalyst must be sufficient to allow the charge‐carriers to migrate to the catalyst surface without recombination.^20^ Reduction in catalyst particle size or the introduction of porosity should in principle promote charge transport to the catalyst surface, and hence improve photocatalytic activity. In practice, defects in small particles can act as recombination sites, and excess porosity may decrease charge carrier mobility to the point that this negates any increase in catalyst surface area.

We first studied a series of *para*‐substituted low molecular weight oligo(phenylene)s (SM1‐SM5). In contrast to previous studies,^21^ we observed a low level of hydrogen evolution (up to around 0.4 μmol h^−1^ with 25 mg of catalyst) under UV irradiation (>295 nm) of a water/methanol/triethylamine mixture without the addition of ruthenium nanoparticles (Figure 1). The hydrogen evolution rate (HER) increases steadily, in an almost linear fashion, with the decrease in optical gap of the oligomers (see insert, Figure 1). This suggests that the increase in HER with oligomer length arises from the red‐shift in optical gap, meaning that more photons are absorbed and more charge carriers are generated for proton reduction and sacrificial donor oxidation. Time‐dependent density functional theory (TD‐DFT) cluster calculations^22^, ^23^ (Supporting Information (SI)) suggest that the thermodynamic driving force for proton reduction is similarly favorable for all oligomers, and largest for SM1.

**Figure 1:**
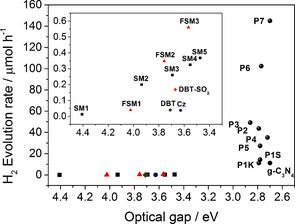
Photocatalytic hydrogen evolution rate (HER) is correlated with the optical gap, both for small molecule oligomers and for conjugated polymers (Cz=9*H*‐carbazole, DBT=dibenzo[*b*,*d*]thiophene). Each measurement was performed with 25 mg catalyst in water/MeOH/triethylamine mixture under broad‐spectrum irradiation (*λ*>295 nm; see Table 1 for visible‐light HERs). HER for platinized g‐C_3_N_4_, as measured on our specific experimental setup. Significantly higher values are known in the literature for g‐C_3_N_4_,^1^ which might be due to differences in the setups used.^29^

Fusing of phenylenes by the introduction of a methylene bridge, or other bridging functionality (Figure 2), lowers the phenylene‐phenylene torsion angle and increases rigidity. For example, fluorene (FSM1) shows a higher degree of conjugation than biphenyl (SM1) due to its more planar conformation.^24^ Extended planarized units in polymers have been shown to decrease the Coulomb binding energy for dissociating electron‐hole pairs and hence to increase exciton dissociation yields.^25^, ^26^ Also, an increase in charge carrier mobility was achieved by planarization in amorphous conjugated polymers.^27^, ^28^

**Figure 2:**
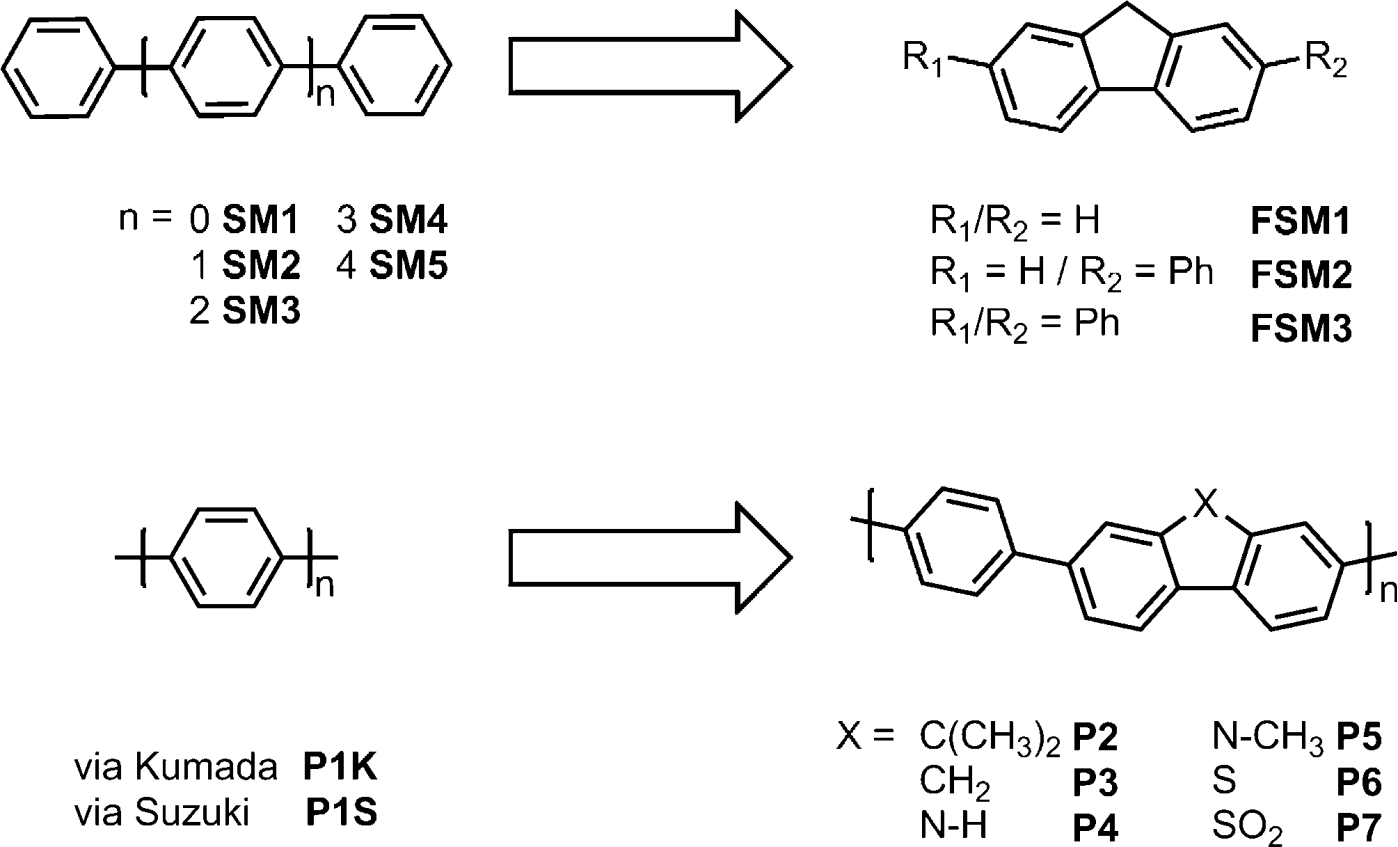
Structures of conjugated phenylene oligomers (SM1–SM5) and poly(*p*‐phenylene)s (P1K, P1S; left) and their planarized fluorene‐type analogs (FSM1–FSM3, P2–P7; right).

For the fluorene oligomers FSM1–FSM3, there is again an almost linear relationship between the hydrogen evolution rate and the decreasing optical gap, much as for the SM1–SM5 oligophenylene series (Figure 1, inset). The optical gaps for FSM1, FSM2, and FSM3 are always red‐shifted relative to their unfused SM equivalents (SM1, SM2, and SM3), in line with the increased conjugation in the fluorene units. However, for pairs of materials with similar optical gaps (e.g., FSM1 and SM2; FSM2 and SM3; FSM3 and SM4), the fluorene materials always produce more hydrogen. This could be because the predicted ionization potentials for the fluorene oligomers are always more negative than those of the equivalent phenylene (e.g., FSM2=−2.75 V; SM3=−2.51 V). These oligomeric model compounds suggest that planarization might be a useful strategy for enhanced photocatalytic water splitting in extended conjugated polymers.

We next synthesized a series of poly(*p*‐phenylene)s (P1K, P1S) and analogous conjugated co‐polymers that incorporated planarized units; that is, fluorenes (P2, P3), carbazoles (P4, P5) and dibenzo[*b*,*d*]thiophene (P6). No solubilizing side chains were incorporated in these polymers, and hence the materials were insoluble in common solvents. Unlike our previous CMP networks,^15^ these linear polymers showed no microporosity. Poly(*p*‐phenylene) P1K was synthesized via Grignard reaction of 1,4‐dibromobenzene with Mg followed by a Kumada‐type reaction. P1S–P6 were all synthesized via Suzuki–Miyaura‐type polycondensation of 1,4‐benzene diboronic acid with the corresponding dibromoarene, using conditions reported previously.^15^ Unlike our previously reported CP‐CMPs,^15^ which were amorphous, all of these linear polymers were found by powder X‐ray diffraction (PXRD) to be semi‐crystalline. For P1K the pattern matches the previously reported structure^30^ and similar patterns were observed for all other polymers. However, no phase‐transitions were observed by differential scanning calorimetry up to 250 °C. All polymers show very similar absorption on‐sets in their UV‐visible reflectance spectra, and hence the optical gaps fall in a narrow range (2.72–2.86 eV; Figure 1 and Table 1). However, the increased conjugation length in the polymers results in a >0.5 eV shift of the onset of absorption and hence the optical gap (Figure 1) compared to the small molecule oligomers (S1–S5; FSM1–FSM3).

**Table 1:** Photophysical properties and hydrogen evolution rates (HERs) for the polymer photocatalysts.

Polymer	Optical gap^[a]^ [eV]	*λ*_em_ [nm]^[b]^	HER >420 nm^[c]^ [μmol h^−1^]	HER >295 nm^[c]^ [μmol h^−1^]
P1K	2.79	456, 483	2.0(±0.1)	10.3(±0.7)
P1S	2.78	452, 478	3.9(±0.2)	14.3(±0.4)
P2	2.79	446	8.3(±0.2)	43.6(±0.2)
P3	2.86	458, 525	0.1(±0.04)	49.2(±0.4)
P4	2.72	434, 452	7.8(±0.2)	35.1(±1.2)
P5	2.78	460	2.2(±0.5)	27.3(±0.4)
P6	2.77	456, 481	26.6(±0.3)	102.4(±0.7)
P7	2.70	477	92.0(±2.0)	145.0(±4.7)

[a] Calculated from the onset of the absorption spectrum, see discussion in the Supporting Information. [b] Photoluminescence emission peak of polymer recorded in the solid state. [c] Reaction conditions: 25 mg polymer was suspended in water/MeOH/triethylamine solution, irradiated by 300 W Xe lamp for 5 hours using a suitable filter.

The photocatalytic activity was tested for these polymers both under broad‐spectrum irradiation (>295 nm; Figure 1, Table 1) and under filtered, visible light (>420 nm; Table 1). Poly(*p*‐phenylene) P1K showed a large increase in HER (10.3 μmol h^−1^) compared to SM5 (0.4 μmol h^−1^) under UV, but lower activity under visible light (>420 nm, 2.0 μmol h^−1^). We propose that the increase in HER of P1K compared to SM5 is not solely due to the change in optical gap because the amount of hydrogen evolved is far larger than expected from the approximately linear trend observed for the oligomers (see Figure 2 inset and SI). Also, the predicted thermodynamic driving force actually *decreases* over the SM1–SM5–P1K series. We propose that this large increase in HER stems from longer charge carrier life‐times in the polymer, due to an increase in conjugation length. The HERs for P1K and P1S were similar (see SI), indicating no influence of the synthesis method on the photocatalytic activity.

The planarized co‐polymers P2–P7 showed greatly enhanced photocatalytic performance compared to poly(*p*‐phenylene)s P1K and P1S. The dimethylfluorene‐*co*‐phenylene polymer, P2, showed a 4‐fold increase in visible‐light activity (>420 nm, 8.3 μmol h^−1^) compared to P1K. Also, the HER under broad‐spectrum irradiation (>295 nm) was significantly enhanced (43.6 μmol h^−1^). The dimethyl side‐chains not only circumvent the problem of keto‐defect formation on the 9‐position,^31^ but also the 9*H*‐fluorene co‐polymer, P3, showed a blue‐shift in absorption resulting in the near complete loss of visible‐light activity (>420 nm, 0.1 μmol h^−1^). However, under broad‐spectrum irradiation (>295 nm, 49.2 μmol h^−1^), P3 showed a similar photocatalytic activity.

The 9*H*‐carbazole polymer, P4, showed similar visible light (7.8 μmol h^−1^) and broad spectrum activity (35.1 μmol h^−1^) when compared with the dimethyl fluorene co‐polymer, P2. A lower performance was also observed for an analogous carbazole polymer, P5, which bears methyl groups (cf., P2 and P3): HERs of 2.2 μmol h^−1^ and 27.3 μmol h^−1^ were found for P5 for visible and broad‐spectrum irradiation, respectively. Again, the lower visible‐light activity can be rationalized by a blue shift in the absorption spectrum. It is possible for both series (P2/P3 and P4/P5) that differences in the chain packing due to the introduction of the methyl groups (see PXRD in the SI), or differences in molecular weight, contribute to this blue‐shift.

The co‐polymer of dibenzo[*b*,*d*]thiophene with benzene‐1,4‐diboronic acid, P6, shows a further increase in hydrogen evolution rate compared to the fluorene co‐polymers (Figure 3). Under broad spectrum irradiation, a rate of 102.4 μmol h^−1^ was observed. More importantly, a greatly improved visible‐light HER (26.6 μmol h^−1^) was achieved for the un‐platinized, as‐synthesized polymer. This hydrogen evolution rate under visible light is almost 20 times higher than for P1K, and a large improvement over platinized commercial g‐C_3_N_4,_ 1 as tested on our setup (>295 nm=11.2 μmol h^−1^; >420 nm=2.7 μmol h^−1^). The UV activity for P6 is comparable to commercial rutile (TiO_2_) loaded with 1 wt. % platinum, as tested on our setup (>295 nm, 107.9 μmol h^−1^, see SI), but unlike P6, rutile shows no visible‐light activity (see SI). The performance of the polymer could be enhanced further by incorporation of the sulfone of dibenzo[*b*,*d*]thiophene. Under broad‐spectrum irradiation, a rate of 145.0 μmol h^−1^ (equivalent to 5800 μmol h^−1^ g^−1^) was observed and under visible light a rate of 92 μmol h^−1^ (3680 μmol h^−1^ g^−1^). This increase is substantial and the increased apparent quantum yields of 1.1 % for P6 and 2.3 % for P7 compared to 0.1 % for P1K reflect this. The incorporation of neither the dibenzo[*b*,*d*]thiophene nor the dibenzo[*b*,*d*]thiophene sulfone unit into P6 and P7 changes the optical gap significantly with respect to P1K and the other planarized co‐polymers, as predicted by (TD‐)DFT calculations (see SI). These calculations also suggest no significant change in the thermodynamic driving force for proton reduction for the various planarized polymers. This supports that neither optical gap nor the thermodynamics of proton reduction can explain these large differences in HER. It is therefore likely that the differences can be ascribed to variations in charge carrier life‐time, charge carrier mobility, specific surface chemistry, or some combination of these factors.

**Figure 3:**
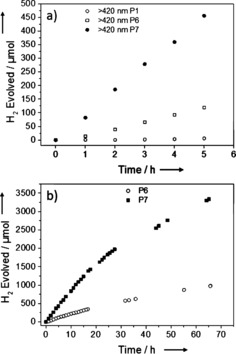
a) Time‐course for photocatalytic H_2_ production using visible light for P1K, P6, and P7 (25 mg catalyst in water/MeOH/triethylamine mixture *λ*>420 nm); b) P6 and P7 (25 mg catalyst in water/MeOH/triethylamine mixture; *λ*>420 nm), photolysis run for a total of 65 h.

Unlike most examples in the literature, no additional metal co‐catalyst was added to these polymers.^15^ However, all of these materials, except P1K, were made using palladium catalysis, and hence significant levels of residual palladium were found by ICP‐OES (see SI). It is therefore possible that residual palladium plays a role in the catalysis, although carbon monoxide poisoning experiments for our earlier CMPs suggested that this was not a major factor for those polymers.^15^ Additionally, the Kumada‐coupled polymer, P1K, displays moderate HERs under broad‐spectrum irradiation (Table 1), and contains no palladium, but a small amount of residual nickel.

Both P6 and P7 show a performance dependency on the wavelength of the light that is used for irradiation. This strong dependency on irradiation wavelength would suggest that these systems, like other semiconductor photocatalysts, will be sensitive to the precise photocatalysts setup,^29^ and in particular the intensity of the photolysis lamp at a given wavelength. For this reason, all of the comparisons made in Table 1 and Figure 1 refer to data collected using exactly the same setup.

P7 was further studied with repeat runs under visible light over a total of 16 hours, with degassing after 10 hours (see SI). The performance is reduced by about 10 % after the 10 hour run, but the material was still active when irradiation was continued for a total of 65 hours, and the overall performance and stability was much better than reported for poly(*p*‐phenylene).^32^ P7 did not show any changes in its UV/vis spectrum, photoluminescence, FT‐IR spectrum, or PXRD pattern after 65 hours of irradiation. Also, a total of 3356 μmol hydrogen was evolved, which is almost more than four times the amount of hydrogen present in the polymer material, ruling out the polymer itself as the hydrogen source.

The ^13^C cross‐polarization (CP) magic angle spinning (MAS) solid state nuclear magnetic resonance (NMR) spectra of both as‐synthesized P6 and P7 polymers, as synthesized, are given in Figures S‐22 and S‐23. All carbon environments have been identified (Table S‐1) confirming the structures given in Figure 2, in agreement with the MALDI‐TOF data. Upon irradiation, the ^13^C CP MAS spectrum of P6 is largely unchanged with respect to as‐synthesized P6, showing that the structure of this polymer is largely unaffected by visible‐light irradiation. The ^13^C CP MAS spectrum of P7 after 33 hours of irradiation is exactly superposable with the spectrum of as‐synthesized P7, demonstrating no chemical change. We note the absence of detectable ^13^C NMR signals in the aliphatic region of the spectrum (20–60 ppm) suggesting that no hydrogenation of the polymer is taking place.^33^

The influence of the hole‐scavenger, or sacrificial donor, was explored for P6. Diethylamine gave a lower performance than triethylamine alone, while methanol alone did not act as a sacrificial donor. A mixture of triethylamine/methanol gave the best photocatalytic performance. This might be due to methanol acting as a co‐solvent that enhances miscibility of the amine with water. Methanol also improves wettability of the polymer particles, and might conceivably contribute to swelling of the hydrophobic polymer. Furthermore, a mixture of Na_2_S/Na_2_SO_3_ in methanol/water showed finite but limited activity with both P6 and P7. Taken together, these data show that hydrogen is evolved via an electron transfer process, and not simply as a result of the decomposition of the hydrogen‐bearing donor or the polymer.

The performance of P6 and P7 under visible light could also be further enhanced to 38.1 and 116 μmol h^−1^, respectively (from 26.6 and 92.0 μmol h^−1^, Table 1) when preformed Pt nanoparticles were added to the reaction mixture prior to irradiation (see SI). By comparison, platinized commercial carbon nitride (Nicanite) had a HER of 2.7 μmol h^−1^, as measured using the same setup.

In summary, planarized conjugated co‐polymers show strongly enhanced hydrogen evolution from water in the presence of a sacrificial electron donor. The co‐polymer of dibenzo[*b*,*d*]thiophene sulfone, P7, shows the highest visible and UV‐activity, even though its absorption profile and optical gap are very similar to poly(*p*‐phenylene). The UV and visible hydrogen evolution rates are greatly improved with respect to our previous best performing CMP material, CP‐CMP10.^15^ This suggests that planarization and rigidification might be a general strategy for improving photocatalytic water splitting activity in organic polymers where the optical gap can be tuned by composition. Our longer‐term goal is to find materials with ionization potentials (HOMO levels) that facilitate overall water splitting using visible light without any sacrificial donors or acceptors.

## Experimental Section

Polymers were prepared using conditions reported previously.^15^ All H_2_ evolution experiments were carried out on exactly the same photolysis setup (see SI) to ensure comparability between the HERs. The carbon nitride control material (Nicanite) was purchased from Carbodeon Ltd Oy and then platinized.^1^ For the AQY experiments H_2_ evolution was measured using a *λ*=420 nm LED as the light source and estimated as described in the SI. The (TD‐)DFT calculations follow the approach developed in Ref. 22. All other details are given in the SI.

## Supporting information

As a service to our authors and readers, this journal provides supporting information supplied by the authors. Such materials are peer reviewed and may be re‐organized for online delivery, but are not copy‐edited or typeset. Technical support issues arising from supporting information (other than missing files) should be addressed to the authors.

Supplementary
