# Supplementary material for: Visible‐Light‐Driven Hydrogen Evolution Using Planarized Conjugated Polymer Photocatalysts
Source: Angew Chem Weinheim Bergstr Ger. 2015 Dec 22;128(5):1824–8. doi: 10.1002/ange.201510542 (PMC4950146; doi:10.1002/ange.201510542)
Supplement: Supplementary file 1 — Supplementary [file ANGE-128-1824-s001.pdf]

Angewandte Chemie
Eine Zeitschrift der Gesellschaft Deutscher Chemiker

Supporting Information

Visible Light-Driven Hydrogen Evolution Using Planarized Conjugated Polymer Photocatalysts

Reiner Sebastian Sprick, Baltasar Bonillo, Rob Clowes, Pierre Guiglion, Nick J. Brownbill, Benjamin J. Slater, Frédéric Blanc, Martijn A. Zwijnenburg, Dave J. Adams, and Andrew I. Cooper**

ange_201510542_sm_miscellaneous_information.pdf

1. Experimental Section

General methods: All reagents were obtained from Sigma-Aldrich or from TCI Europe and used as received, except for 2,7-dibromo-*N*-methylcarbazole, 2,7-dibromo-9,9-dimethylfluorene, and 3,7-dibromodibenzo[*b*,*d*]thiophene 5,5-dioxide which were synthesized using literature procedures.[1,2] Graphitic carbon nitride (Nicanite) was obtained from Carbodeon Ltd Oy and used as received. Water for the hydrogen evolution experiments was purified using an ELGA LabWater system with a Purelab Option S filtration and ion exchange column (ρ = 15 MΩ cm) without pH level adjustment. Reactions were carried out under nitrogen atmosphere using standard Schlenk techniques. ^{1}H and ^{13}C NMR spectra were recorded on Bruker Avance 400MHz NMR in $CHCl_3$ at 25 ºC. $^{13}C\{^{1}H\}$ NMR Spectra were recorded at 100 MHz in $CHCl_3$ at 25 ºC. All spectra are reported in ppm and are referenced to the residual solvent peak. Mass spectroscopy was performed using an Agilent QTOF 7200 Spectrometer. CHN Analysis was performed on a Thermo EA1112 Flash CHNS-O Analyzer using standard microanalytical procedures. Palladium content was determined *via* ICP-OES by Butterworth Laboratories Ltd (Teddington, United Kingdom). Transmission FT-IR spectra were recorded on a Bruker Tensor 27 at room temperature; samples were prepared as pressed KBr pellets. Thermogravimetric analysis was performed on an EXSTAR6000 by heating samples at 10 °C min^{-1} under air in open aluminium pans from 40 to 600 °C and holding at 600 °C for 30 minutes. DSC analysis was performed on a TA Instruments DSC Q2000 in an open pan under nitrogen. The samples were heated at 10 ºC min^{-1} from -30 ºC to 150 ºC, held for 2 min, and then cooled to -30 ºC at 10 ºC min^{-1}. After being held at -30 ºC for 2 min, the samples were heated to 250 ºC at 10 ºC min^{-1}, held again for 2 min, and cooled to -30 ºC at 10 ºC min^{-1}. Finally, the samples were heated again to 250 ºC at 10 ºC min^{-1}. The UV-Visible absorption spectra of the polymer networks were recorded on a Shimadzu UV-2550 UV-Vis spectrometer as powders in the solid state, $CHCl_3$ solution or in PEG-400 suspension. The fluorescence spectra of the polymer powders were measured with a Shimadzu RF-5301PC fluorescence spectrometer at room temperature or in $CHCl_3$ solutions with an absorbance lower than 0.1. A USB2000+XR1-es with a QP400-1-UV-VIS-fiber optic probe was used to record the emission spectra of the lamp and of the lamp using coloured glass filters. The probe was placed 10 cm in front of the filter holder. The integration time was 35 ms and 100 scans were recorded and an average was taken. Imaging of the polymer morphology was achieved on a Hitachi S4800 Cold Field Emission SEM, with secondary electron, backscatter and transmission detectors. EDX measurements were performed on an Oxford Instruments

INCA ENERGY 250 M/X. Matrix-assisted laser desorption/ionization time-of-flight mass spectrometry (MALDI-TOF MS) was performed on a Shimadzu Biotech AXIMA Confidence MALDI mass spectrometer in linear (positive) mode, referencing against a PEG standard (4 kDa). Polymer samples (5 mg) were suspended in THF (5 mL) by ultrasonication for 30 min and 40 μL of the suspension were mixed with 20 μL of a 30 mg mL^{-1} solution of 8-dihydroxy-9,10-dihydroanthracen-9-one in THF. One drop of this mixed suspension was then spotted onto a target plate which had been pre-spotted with a drop of 10 mg mL^{-1} solution of NaI in THF. The MALDI plate was allowed to dry for 10 min at room temperature before the measurement. PXRD measurements were performed on a PANalytical X'Pert PRO MPD, with a Cu X-ray source, used in high throughput transmission mode with Kα focusing mirror and PIXCEL 1D detector. All solid-state NMR experiments were performed on a 400 MHz 9.4 T Bruker Avance III HD solid-state NMR spectrometer equipped with a 4 mm HXY triple-resonance magic angle spinning (MAS) probe (in double resonance mode) with the ^{1}H channel tuned to ^{1}H at $\nu_0(^{1}H)$ = 400.13 MHz and the X channel tuned to ^{13}C at $\nu_0(^{13}C)$ = 100.03 MHz. Experiments were performed at room temperature under MAS at ν_r = 6, 7 or 10 kHz. ^{1}H pulses and SPINAL-64 heteronuclear decoupling[3] were performed at a radio-frequency (rf) field amplitude of 83 kHz. ^{1}H ^{13}C cross polarization (CP) MAS experiments were obtained with a ^{13}C rf field of 60 kHz, while the ^{1}H rf field amplitude was ramped to obtain maximum signal at a ^{1}H rf field of approximately 60 kHz, and a contact time of 2 ms. For ^{13}C spectra, 2048-4096 scans were accumulated with a 3 s recycle delay. The ^{13}C chemical shifts were referenced to the CH carbon of adamantane at 29.45 ppm.[4] Samples were packed in a zirconia rotor with a KelF cap (samples for NMR spectra recorded at ν_r = 6 and 7 kHz spectra were centre packed with a PTFE plug), and NMR data were obtained and analysed using TopSpin 3.2.

Hydrogen evolution experiments: A flask was charged with the polymer powder (25 mg), water (7.5 mL), triethylamine (7.5 mL), methanol (7.5 mL), and sealed with a septum. The resulting suspension was ultrasonicated until the photocatalyst was dispersed before degassing by N_2 bubbling for 30 minutes. The reaction mixture was illuminated in a 90° angle with a 300 W Newport Xe light-source (Model: 6258, Ozone free) for the time specified. The lamp was cooled by water circulating through a metal jacket. Gas samples were taken with a gas-tight syringe, and run on a Bruker 450-GC gas chromatograph equipped with a Molecular Sieve 13X 60-80 mesh 1.5 m × ⅛" × 2 mm ss column at 50 °C with an argon flow of 40.0 mL min^{-1}. Hydrogen was detected with a thermal conductivity

detector referencing against standard gas with a known concentration of hydrogen. Hydrogen dissolved in the reaction mixture was not measured and the pressure increase generated by the evolved hydrogen was neglected in the calculations. The rates were determined from a linear regression fit and the error is given as the standard deviation of the amount of hydrogen evolved. No hydrogen evolution was observed for a mixture of water/methanol/trimethylamine under >295 nm illumination in absence of a photocatalyst.

Synthesis of 2-phenyl-9*H*-fluorene: 2-Bromofluorene (1.85 g, 7.54 mmol), phenylboronic acid (1.38 g, 11.31 mmol), toluene (50 mL), K_2CO_3 solution (25 mL, 2 M) and Starks' catalyst (2 drops) were combined. After degassing of the reaction mixture for 30 min by bubbling with N_2 $Pd(PPh_3)_4$ (130 mg, 1.5 mol%) was added. After further degassing for 5 min, the reaction mixture was heated to 120 °C for 2 days. After cooling to room temperature, phases were separated and the aqueous phase extracted with ethyl acetate. The combined organic phases were dried over $MgSO_4$, filtered, and the solvent was evaporated. The crude product was purified by column chromatography (hexane, gradient to hexane:ethyl acetate 7:3 on SiO_2) to give the product as a white powder (1.58 g, 86%). ^{1}H NMR (400 MHz, $CDCl_3$) δ 7.85 (d, J = 8.0 Hz, 1H), 7.82 (d, J = 7.5 Hz, 1H), 7.78 (d, J = 1.0 Hz, 1H), 7.70 – 7.60 (m, 3H), 7.57 (d, J = 7.0 Hz, 1H), 7.50 – 7.43 (m, 2H), 7.43 – 7.29 (m, 3H), 3.97 (s, 2H). $^{13}C\{^1H\}$ NMR (100 MHz, $CDCl_3$) δ 144.0, 143.6, 141.7, 141.6, 141.1, 140.0, 128.9, 127.3, 127.3, 127.0, 126.9, 126.2, 125.2, 124.0, 120.3, 120.1, 37.2. Anal. Calcd for $C_{19}H_{14}$: C, 94.18; H, 5.82%; Found C, 94.25; H, 5.83%. HRMS (CI, CH_4): m/z Calcd for $C_{19}H_{14}$: 243.1175 $(M)^+$; found: 243.1168.

Synthesis of 2,7-diphenyl-9*H*-fluorene: 2,7-Dibromofluorene (1.22 g, 3.77 mmol), phenylboronic acid (1.38 g, 11.31 mmol), toluene (50 mL), K_2CO_3 solution (25 mL, 2 M) and Starks' catalyst (2 drops) were combined. After degassing of the reaction mixture for 30 min by bubbling with N_2 $Pd(PPh_3)_4$ (130 mg, 3 mol%) was added. After further degassing for 5 min, the reaction mixture was heated to 120 °C for 2 days. After cooling to room temperature phases were separated and the aqueous phase extracted with chloroform. The combined organic phases were dried over $MgSO_4$, filtered over a short silica plug, and the solvent was evaporated. The crude product was purified by recrystallization from toluene and washing of the crystals with cold methanol to give the product as a white powder (0.89 g, 74%). ^{1}H NMR (400 MHz, $CDCl_3$) δ 7.87 (d, J = 7.9 Hz, 1H), 7.80 (d, J = 0.9 Hz, 1H), 7.70 – 7.61 (m, 3H), 7.50 – 7.43 (m, 2H), 7.39 – 7.33 (m, 1H), 4.03 (s, 1H). $^{13}C\{^1H\}$ NMR (100 MHz, $CDCl_3$) δ 144.2, 141.5, 140.6, 139.9, 128.8, 127.2, 127.2, 126.1, 123.8, 120.2,

37.1. Anal. Calcd for $C_{25}H_{18}$: C, 94.30; H, 5.70%; Found C, 94.16; H, 5.66%. HRMS (CI, CH_4): m/z Calcd for $C_{25}H_{18}$: 319.1485 $(M)^+$; found: 319.1481.

Synthesis of P1K (Kumada-type polycondensation): 1,4-Dibromobenzene (11.80 g, 50.44 mmol), magnesium (1.22 g, 50.20 mmol) and THF (80 mL, anhydrous) were heated to reflux for 45 min. [1,3-Bis(diphenylphosphino)propane]dichloronickel(II) (200 mg, 0.4 mmol) were added and the reaction was kept at reflux for 22 hours. After cooling the crude polymer was poured into acetone, filtered and washed with hydrochloric acid (1 M), water, methanol and THF. Further purification was performed by Soxhlet extraction with methanol and THF. After drying under reduced pressure the product were obtained as a light green powder (3.76 g, 98%). Anal. Calcd for $(C_6H_4)_n$: C, 94.70; H, 5.30%; Found C, 87.54; H, 5.15%, Ni, 0.049%.

General procedure for the synthesis of polymers P2–P6 (Suzuki-Miyaura-type polycondensation): A flask was charged with the monomers, *N*,*N*-dimethylformamide, an aqueous solution of K_2CO_3 (2.0 M), and $Pd(PPh_3)_4$. The mixture was degassed by bubbling with N_2 for 30 minutes and heated to 150 °C for 48 hours. The mixture was cooled to room temperature and poured into water. The precipitate was collected by filtration and washed with H_2O and methanol. Further purification of the polymers was carried out by Soxhlet extraction to remove any low-molecular weight by-products and the product was dried under reduced pressure. MALDI-TOF MS analysis showed the presence of the repeat unit for all polymers up to m/z = 3000 Da. In all cases, the main series of MALDI-TOF peaks was H/H terminated; minor series bearing either phenyl or the arene co-monomer as end-groups were also present, and debromination seems to occur either during the reaction or during work-up. No fragments corresponding to homocoupling were observed. The MALDI-TOF data confirm that the polymerizations all occurred as expected, but do not provide a quantitative measure of molecular weight for these insoluble polymers.[5] Thermogravimetric analysis indicates good thermal stability of the materials up to at least 350 °C.

Synthesis of P1S: 1,4-Dibromobenzene (1.18 g, 5.0 mmol), 1,4-benzene diboronic acid (0.829 g, 5.0 mmol), $Pd(PPh_3)_4$ (38 mg, 0.7 mol%), *N*,*N*-dimethylformamide (75 mL) and aqueous K_2CO_3 (2.0 M, 15 mL) were used in this reaction. After work-up and Soxhlet extraction with THF, the product was obtained as a green-grey powder (588 mg, 77%). Anal. Calcd for $(C_{12}H_8)_n$: C, 94.70; H, 5.30%; Found C, 83.43; H, 4.94%, Pd, 0.33%.

Synthesis of P2: 2,7-Dibromo-9,9-dimethyl-fluorene (0.704 g, 2.0 mmol), 1,4-benzene diboronic acid (0.332 g, 2.0 mmol), $Pd(PPh_3)_4$ (15 mg, 0.65 mol%), *N*,*N*-dimethylformamide (30 mL) and aqueous K_2CO_3 (2.0 M, 6 mL) were used in this reaction. After work-up and Soxhlet extraction with THF, the product was obtained as a light-green powder (416 mg, 78%). Anal. Calcd for $(C_{21}H_{16})_n$: C, 93.99; H, 6.01%; Found C, 82.43; H, 5.41%, Pd, 0.53%.

Synthesis of P3: 2,7-Dibromo-9*H*-fluorene (0.324 g, 1.0 mmol), 1,4-benzene diboronic acid (0.165 g, 1.0 mmol), $Pd(PPh_3)_4$ (15 mg, 1.3 mol%), *N*,*N*-dimethylformamide (15 mL) and aqueous K_2CO_3 (2.0 M, 3 mL) were used in this reaction. After work-up and Soxhlet extraction with THF, the product was obtained as a light green-yellow powder (154 mg, 64%). Anal. Calcd for $(C_{19}H_{12})_n$: C, 94.97; H, 5.03%; Found C, 88.82; H, 4.92%, Pd, 0.45%.

Synthesis of P4: 2,7-Dibromo-9*H*-carbazole (0.325 g, 1.0 mmol), 1,4-benzene diboronic acid (0.165 g, 1.0 mmol), $Pd(PPh_3)_4$ (15 mg, 1.3 mol%), *N*,*N*-dimethylformamide (15 mL) and aqueous K_2CO_3 (2.0 M, 3 mL) were used in this reaction. After work-up and Soxhlet extraction with THF, the product was obtained as a green powder (145 mg, 60%). Anal. Calcd for $(C_{18}H_{11}N)_n$: C, 89.60; H, 4.60; N, 5.81%; Found C, 80.52; H, 4.47; N, 4.83%, Pd, 0.61%.

Synthesis of P5: 2,7-Dibromo-9-methyl-carbazole (0.170 g, 0.5 mmol), 1,4-benzene diboronic acid (0.083 g, 0.5 mmol), $Pd(PPh_3)_4$ (7 mg, 1.2 mol%), *N*,*N*-dimethylformamide (10 mL) and aqueous K_2CO_3 (2.0 M, 2 mL) were used in this reaction. After work-up and Soxhlet extraction with chloroform, the product was obtained as a green powder (80 mg, 63%). Anal. Calcd for $(C_{19}H_{13}N)_n$: C, 89.38; H, 5.13; N, 5.49%; Found C, 75.19; H, 4.48; N, 3.92%, Pd, 0.58%.

Synthesis of P6: 3,7-Dibromodibenzo[*b*,*d*]thiophene (0.684 g, 2.0 mmol), 1,4-benzene diboronic acid (0.331g, 2.0 mmol), $Pd(PPh_3)_4$ (40 mg, 1.7 mol%), *N*,*N*-dimethylformamide (40 mL) and aqueous K_2CO_3 (2.0 M, 8 mL) were used in this reaction. After work-up and Soxhlet extraction with chloroform, the product was obtained as a light green powder (390 mg, 75%). Anal. Calcd for $(C_{18}H_{10}S)_n$: C, 83.69; H, 3.90; S, 12.41%; Found C, 79.10; H, 3.86; S, 11.56%, Pd, 0.60%.

Synthesis of P7: 3,7-dibromodibenzo[*b*,*d*]thiophene 5,5-dioxide (0.748 g, 2.0 mmol), 1,4-benzene diboronic acid (0.331 g, 2.0 mmol), $Pd(PPh_3)_4$ (20 mg, 0.9 mol%), *N*,*N*-dimethylformamide (40 mL) and aqueous K_2CO_3 (2.0 M, 8 mL) were used in this reaction.

After work-up and Soxhlet extraction with chloroform, the product was obtained as a green powder (559 mg, 96%). Anal. Calcd for $(C_{18}H_{10}SO_2)_n$: C, 74.46; H, 3.47; S, 11.04%; Found C, 67.67; H, 3.38; S, 9.90%, Pd, 0.38%.

2. UV and Photoluminescence Spectra

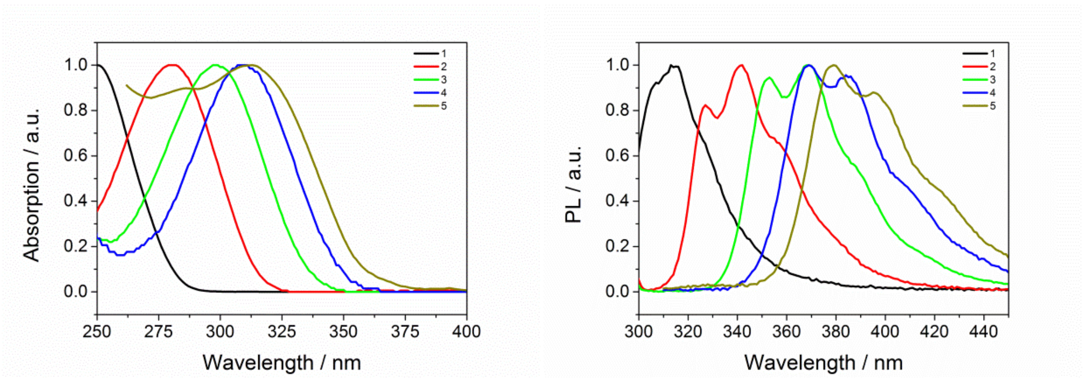

Figure S-1. UV-Vis and photoluminescence spectra (λ_{exc}= 275 nm) of **SM1–SM5** in $CHCl_3$ solution.

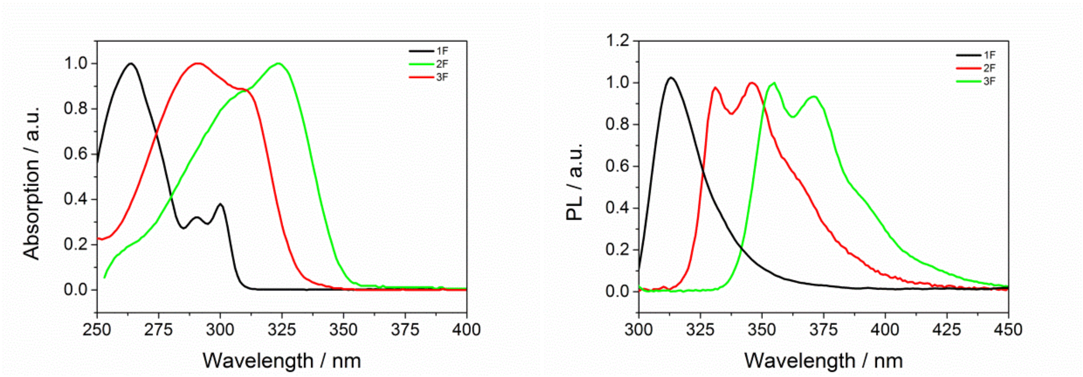

Figure S-2. UV-Vis and photoluminescence spectra (λ_{exc}= 275 nm) of **FSM1–FSM3** in $CHCl_3$ solution.

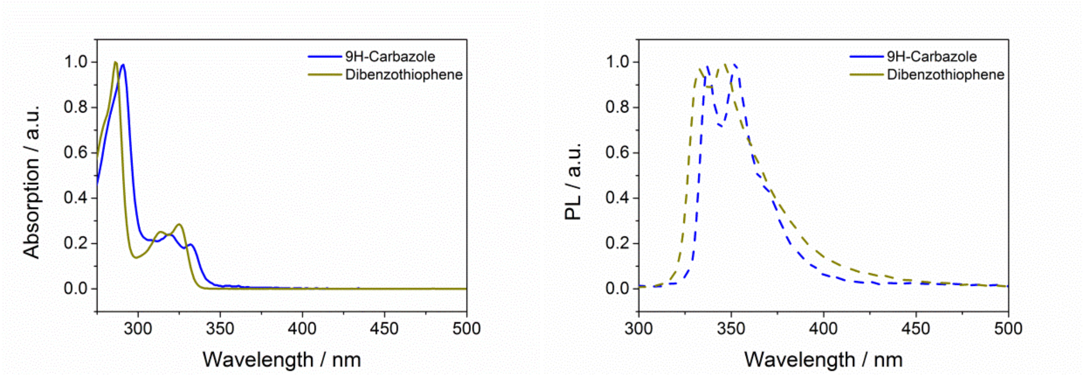

Figure S-3. UV-Vis and photoluminescence (λ_{exc}= 275 nm) spectra of **9*H*-carbazole** and **dibenzo[*b*,*d*]thiophene** in $CHCl_3$ solution.

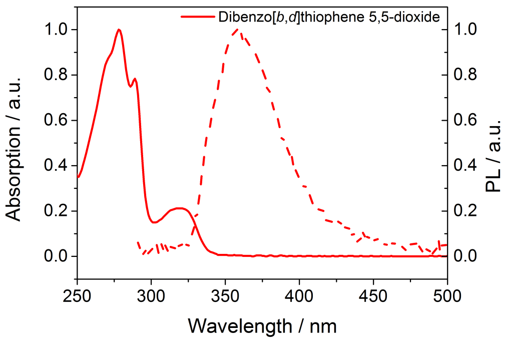

Figure S-4. UV-Vis and photoluminescence spectra (λ_{exc}= 275 nm) of **dibenzo[*b*,*d*]thiophene 5,5-dioxide** in $CHCl_3$ solution.

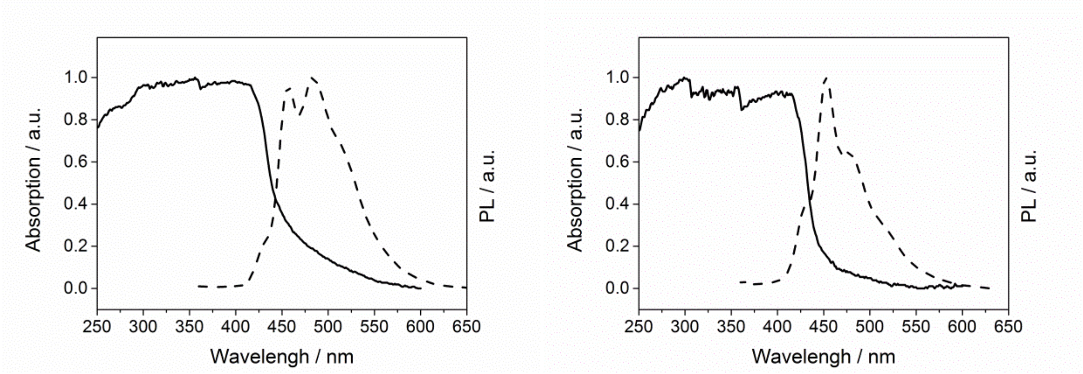

Figure S-5. Solid-state UV-Vis spectrum (solid line) and photoluminescence spectrum (λ_{exc} = 360 nm dashed line) of **P1K** (left) and **P1S** (right).

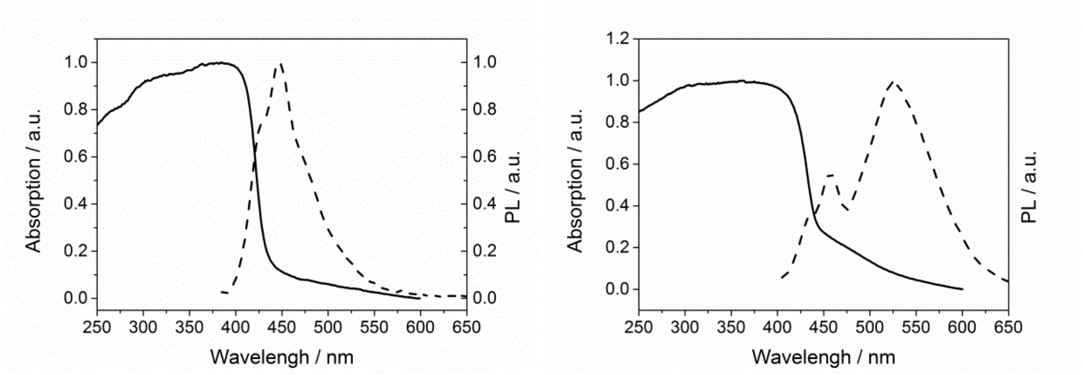

Figure S-6. Solid-state UV-Vis spectrum (solid line) and photoluminescence spectrum (λ_{exc}= 360 nm, dashed line) of **P2** (left) and **P3** (right).

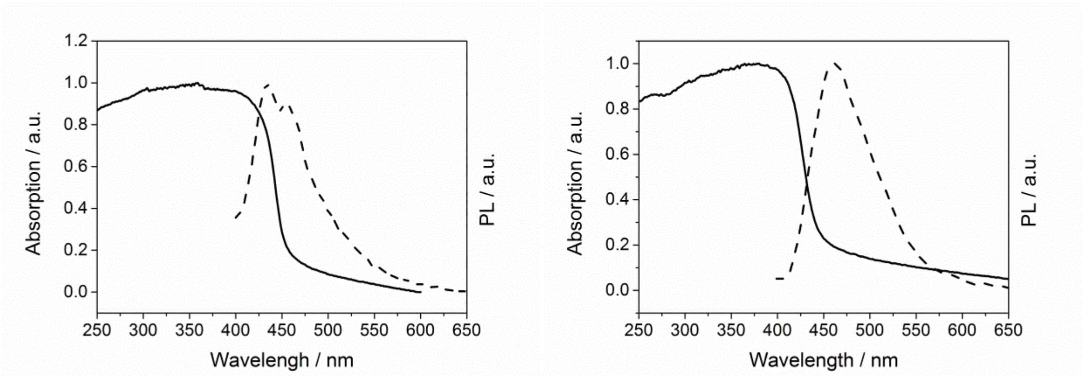

Figure S-7. Solid-state UV-Vis spectrum (solid line) and photoluminescence spectrum (λ_{exc}= 360 nm, dashed line) of **P4** (left) and **P5** (right).

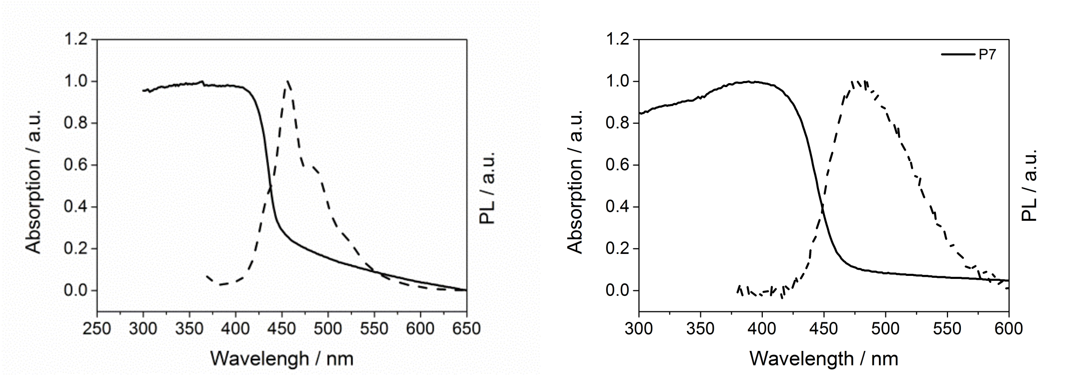

Figure S-8. Solid-state UV-Vis spectrum (solid line) and photoluminescence spectrum (λ_{exc}= 360 nm, dashed line) of **P6** (left) and **P7** (right).

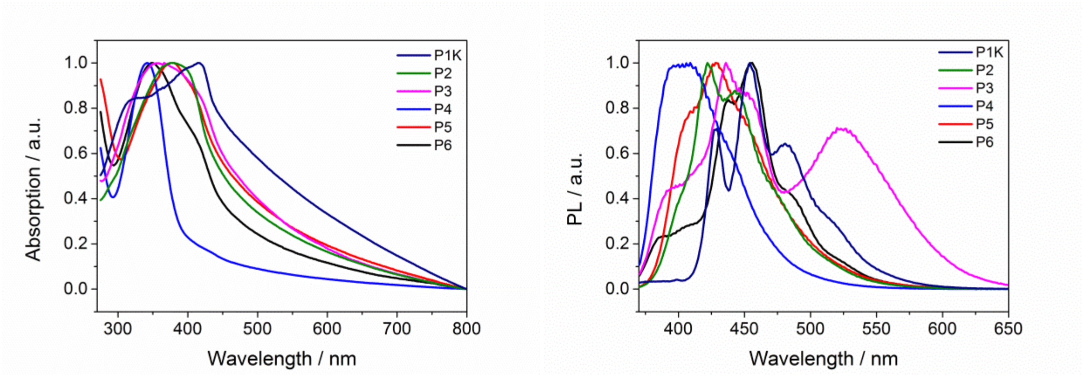

Figure S-9. UV-Vis spectra (left) and photoluminescence spectra (λ_{exc}= 360 nm, right) of **P1K**–**P6** in PEG-suspension.

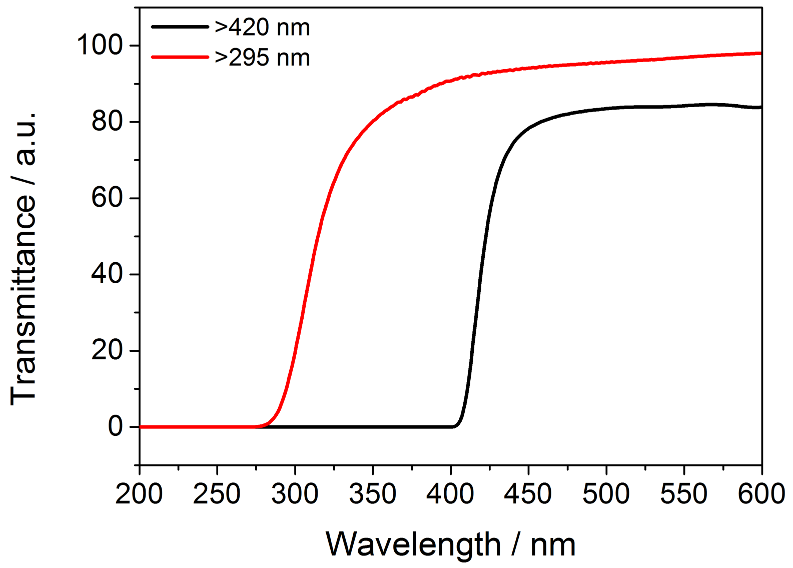

Figure S-10. Transmittance characteristics of the >295 nm and >420 nm filter used in this work.

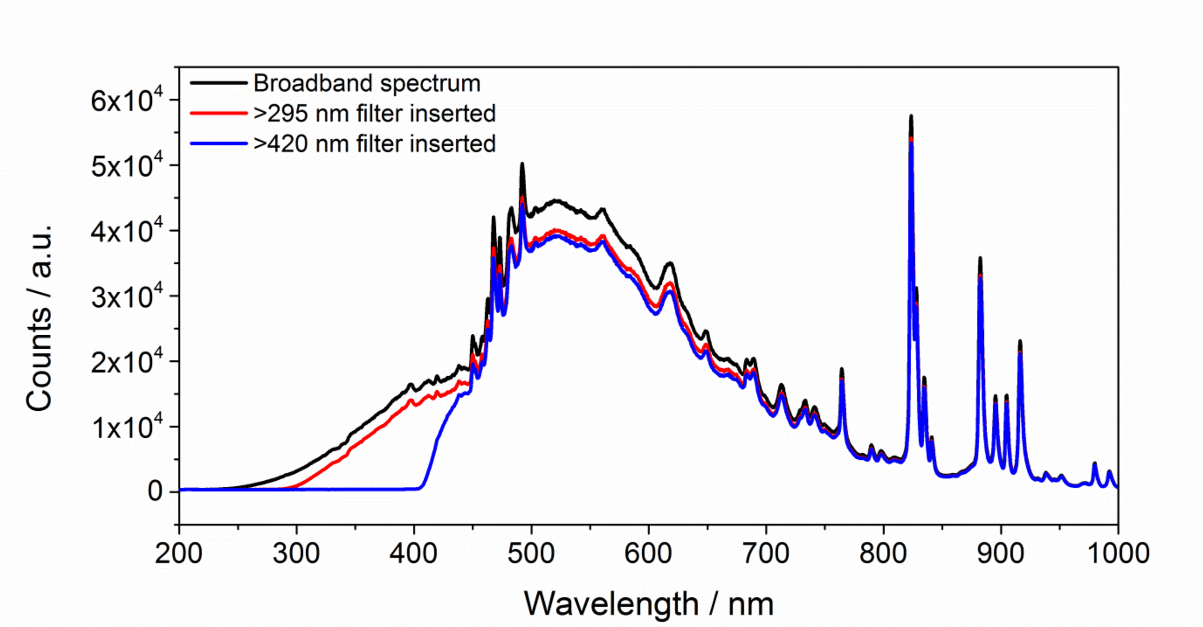

Figure S-11. Unfiltered, broadband output profile of the 300 W Xe-lamp, and output with >295 nm and >420 nm filters, respectively, inserted in front of the lamp.

3. Fourier Transform Infrared Spectroscopy

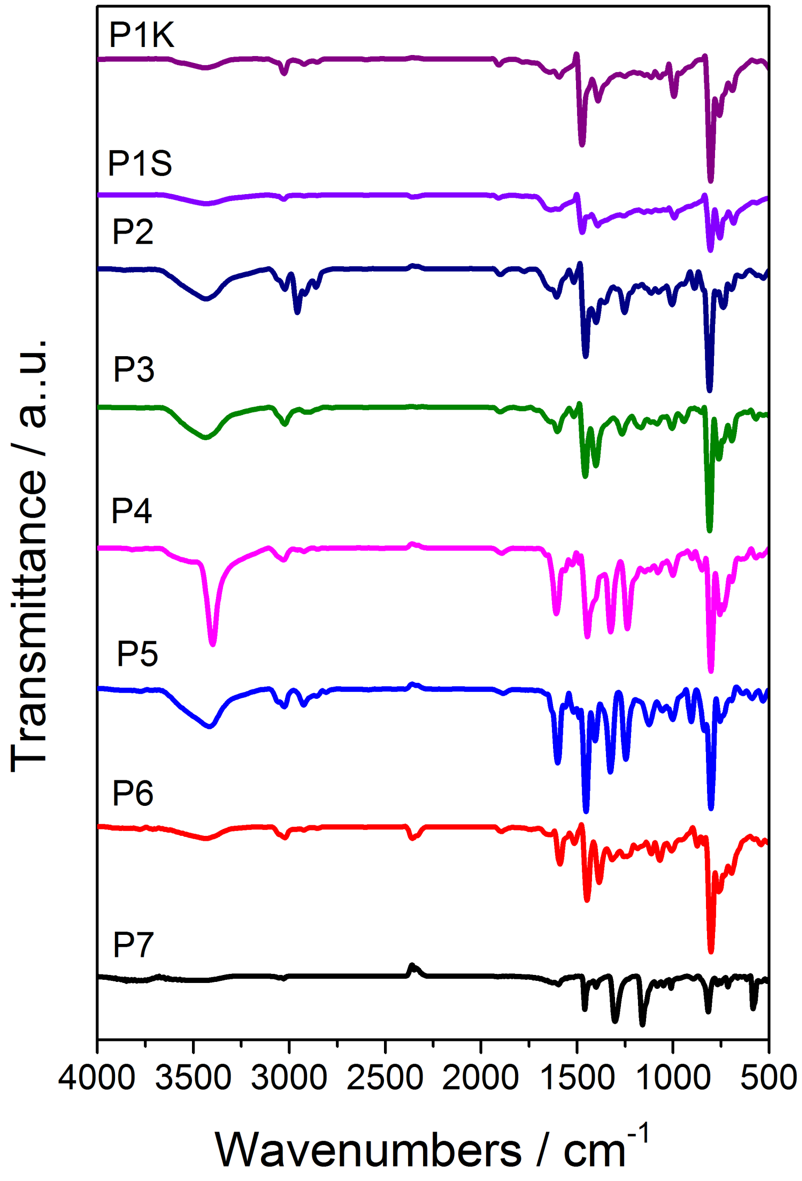

Figure S-12. Transmission FT-IR spectra of polymers **P1K**, **P1S**-**P7** as KBr pellets.

4. MALDI-TOF MS

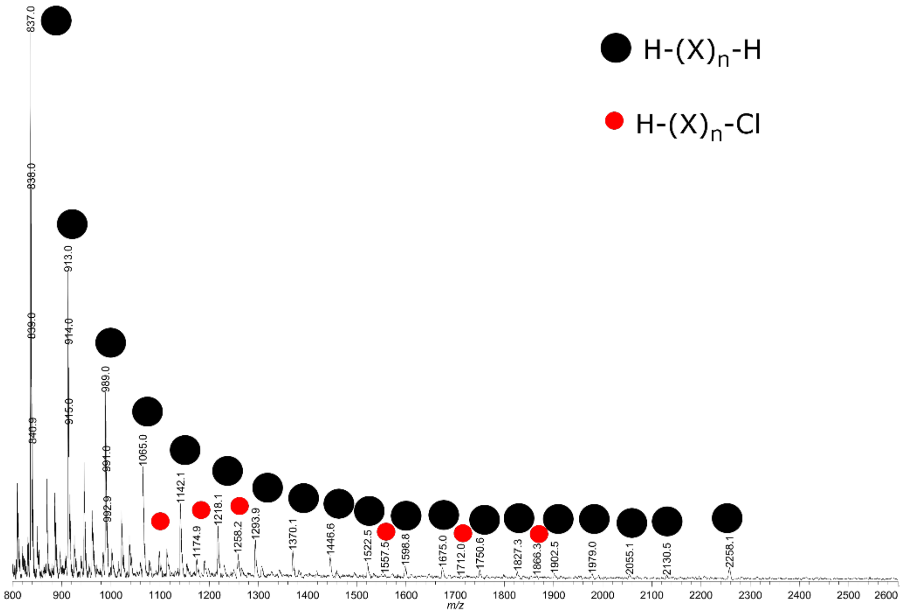

Figure S-13. MALDI-TOF MS spectrum of **P1K**. X = C_6H_4.

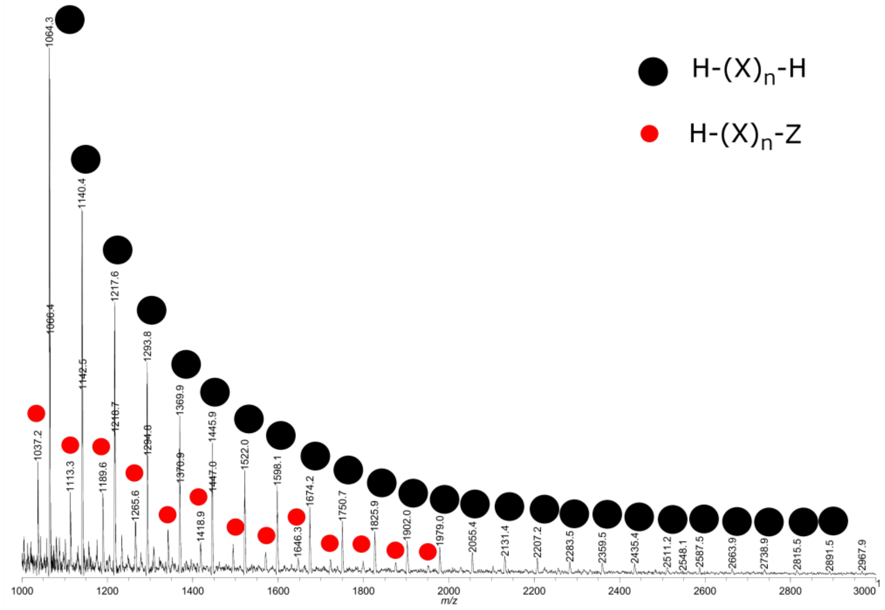

Figure S-14. MALDI-TOF MS spectrum of **P1S**. X = C_6H_4. Besides the H/H-terminated main-series a series with the repeat unit of the polymer (m/z = 76 Da) and unknown end-groups with the mass of m/z = 49 Da were identified.

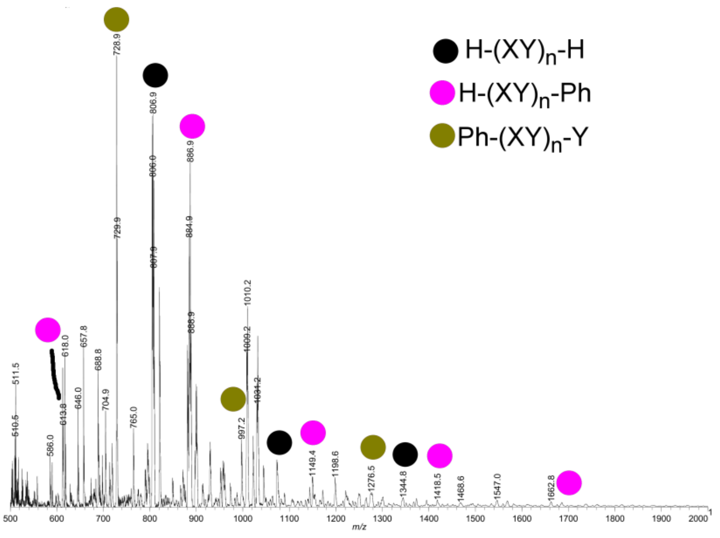

Figure S-15. MALDI-TOF MS spectrum of **P2**. X = C_6H_4, Y = dimethylfluorene.

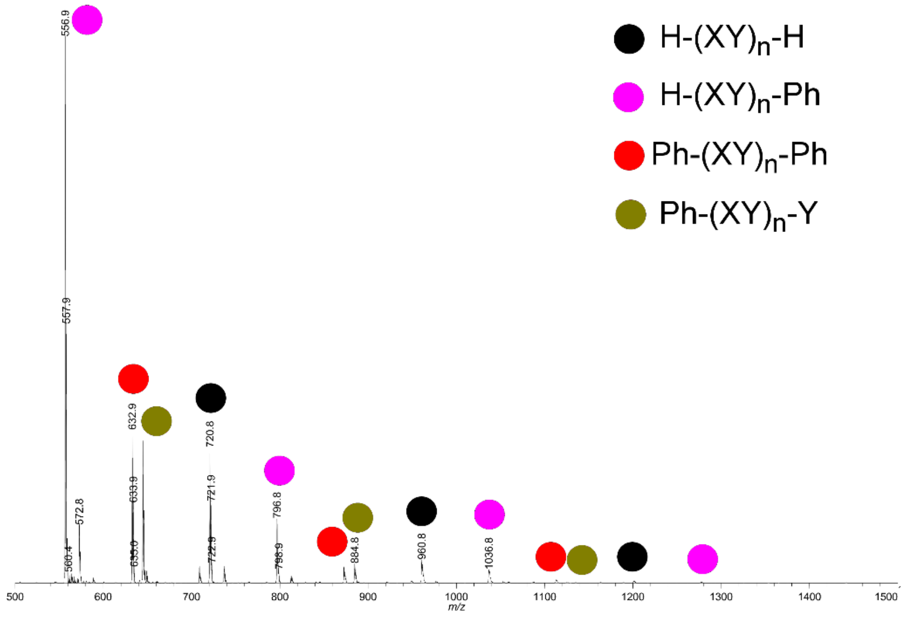

Figure S-16. MALDI-TOF MS spectrum of **P3**. X = C_6H_4, Y = fluorene.

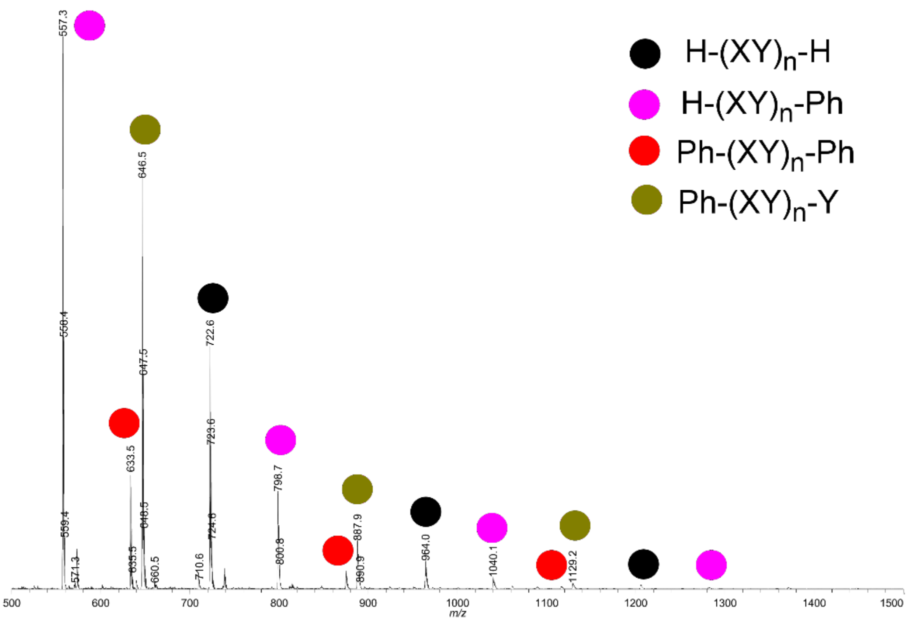

Figure S-17. MALDI-TOF MS spectrum of **P4**. X = C_6H_4, Y = 9*H*-carbazole.

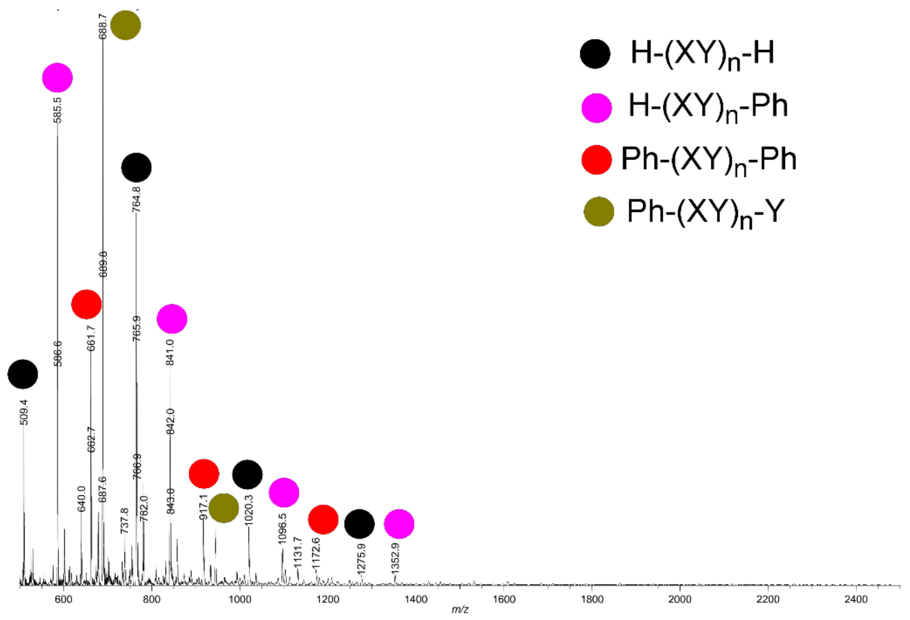

Figure S-18. MALDI-TOF MS spectrum of **P5**. X = C_6H_4, Y = 9-methyl-carbazole.

.

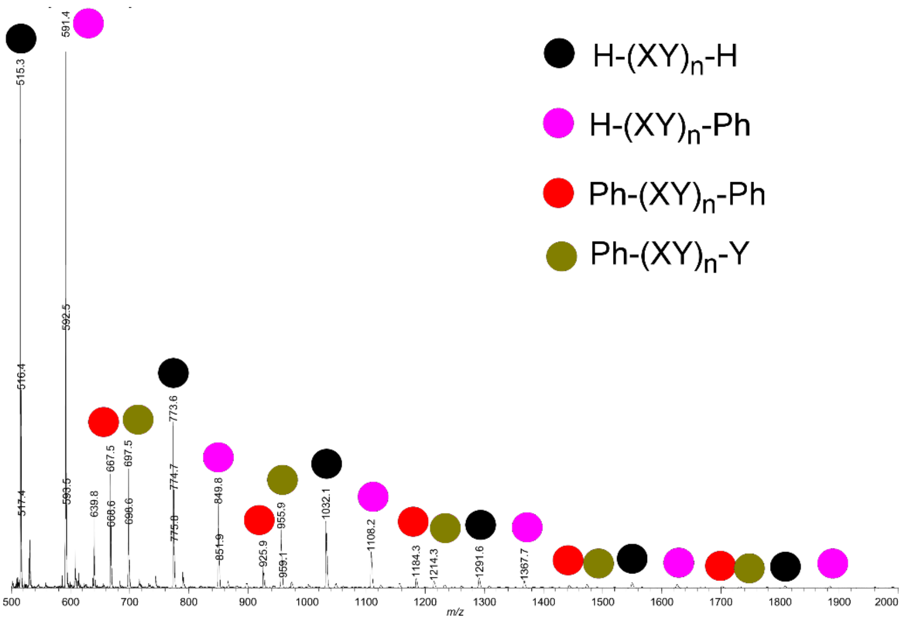

Figure S-19. MALDI-TOF MS spectrum of **P6**. X = C_6H_4, Y = dibenzo[*b*,*d*]thiophene.

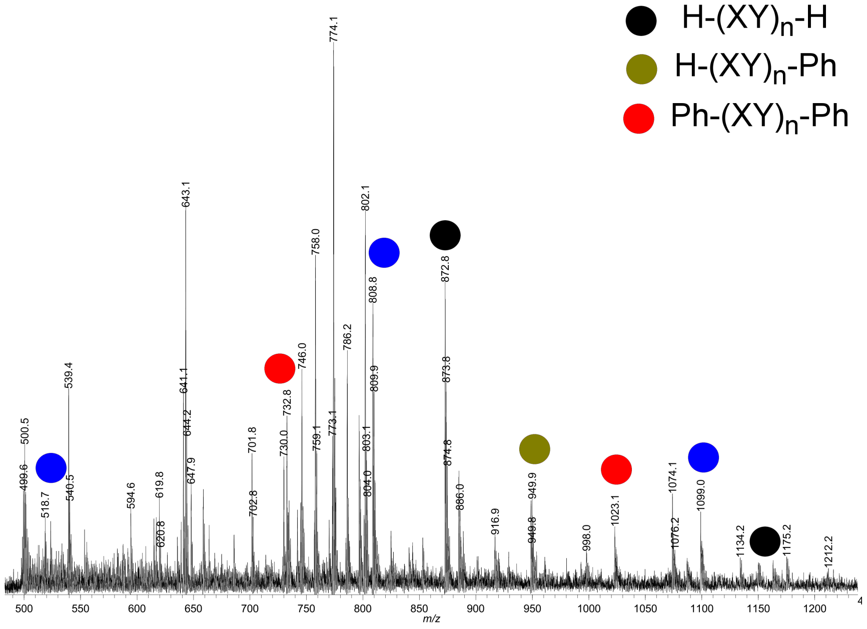

Figure S-20. MALDI-TOF MS spectrum of **P7**. X = C_6H_4, Y = dibenzo[*b*,*d*]thiophene sulfone.

5. Solid-State NMR

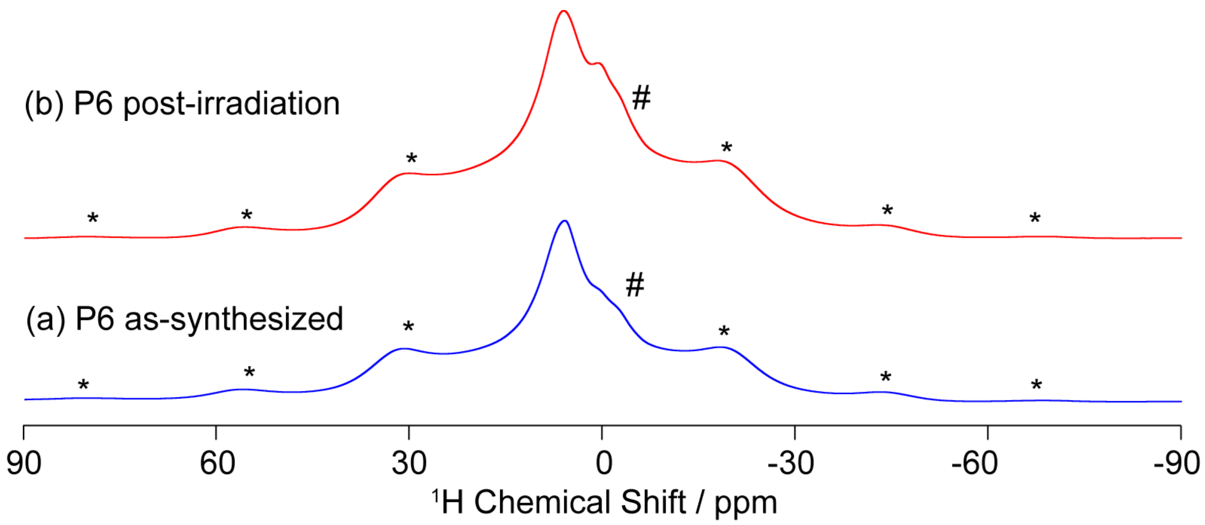

Figure S-21. ^{1}H MAS NMR of P6 (a) as-synthesized and (b) after 38 hours of irradiation. Both spectra were obtained at ν_r = 10 kHz. Asterisks (*) denote spinning sidebands, hashes (#) denote probe background signal.

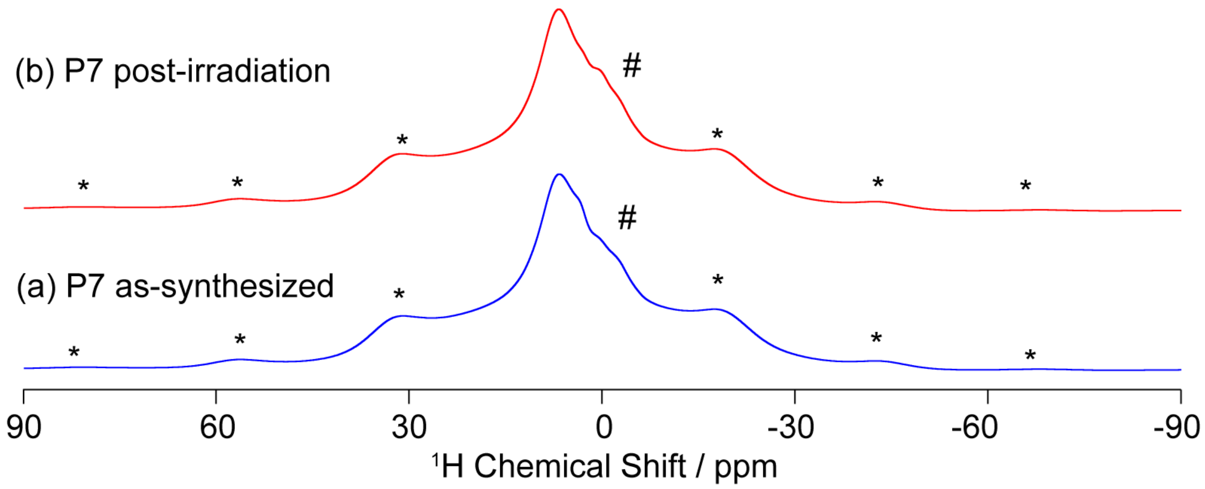

Figure S-22. ^{1}H MAS NMR of P7 (a) as-synthesized and (b) after 33 hours of irradiation. Spectra were obtained at ν_r = 10 kHz. Asterisks (*) denote spinning sidebands, hashes (#) denote probe background signal.

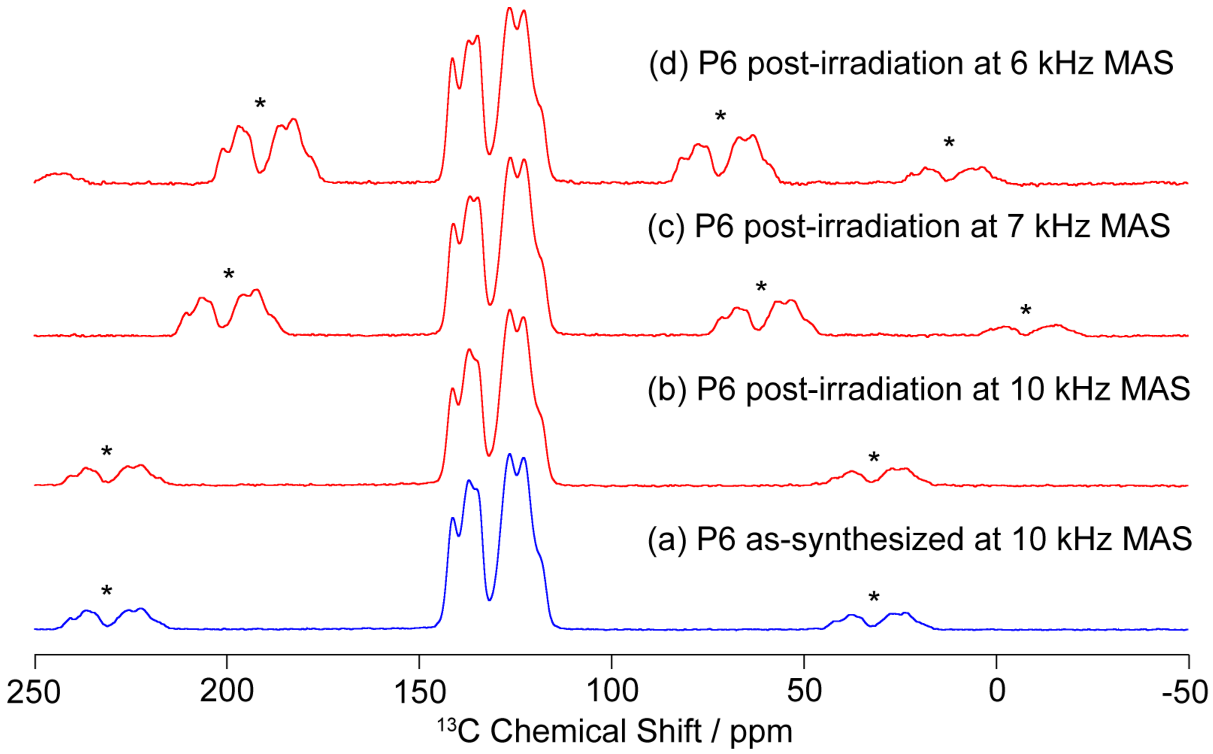

Figure S-23. ^{13}C CP MAS NMR of P6 (a) as-synthesized and (b), (c) and (d) after 38 hours of irradiation, at various MAS rates. Asterisks (*) denote spinning sidebands.

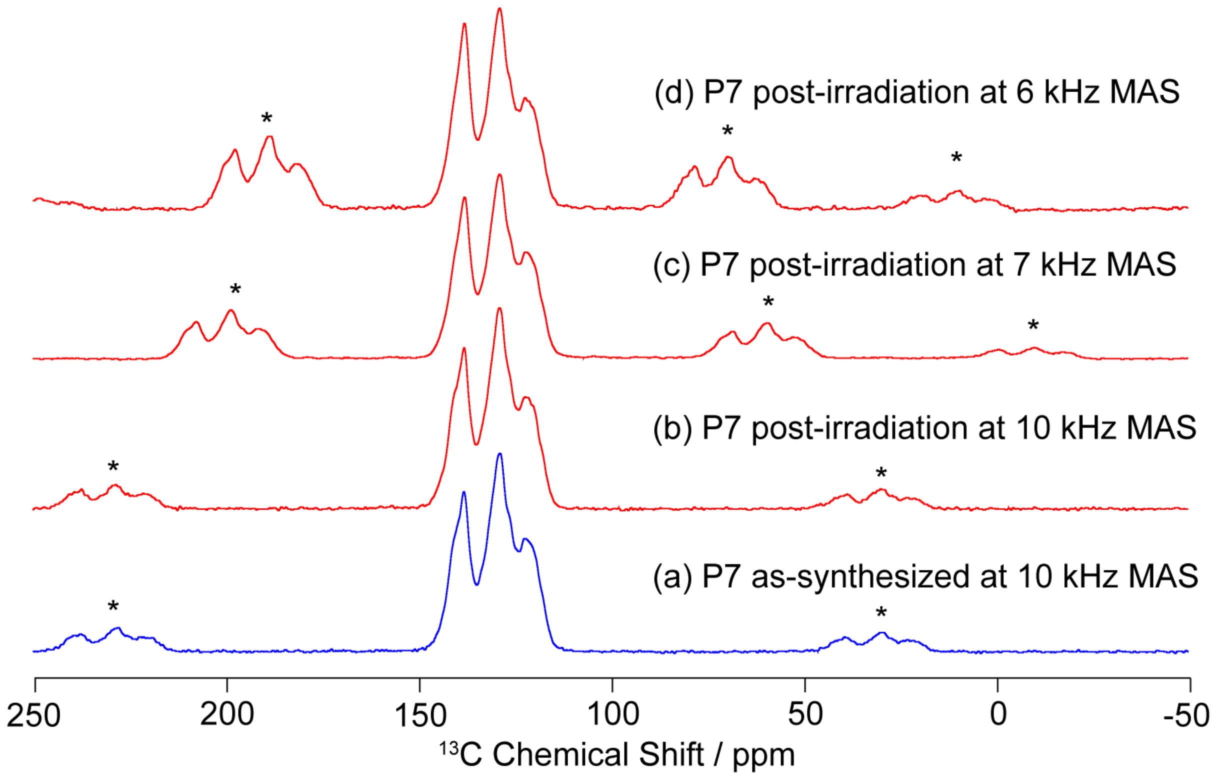

Figure S-24. ^{13}C CP MAS NMR of P7 (a) as-synthesized and (b), (c) and (d) after 33 hours of irradiation, at varied MAS rates. Asterisks (*) denote spinning sidebands.

Polymer	δ_{iso} / ppm	^{13}C Assignments
**P6**	119	-SCC***C***H-
	123	-SC***C***H-
	127	-***C***H$_{(Ph)}$-, SCCCH***C***H-
	135	-SC***C***-
	137	-***C***$^{(IV)}_{(Ph)}$-, -SCCH***C***-
	141	-S***C***-
**P7**	119	-SCC***C***H-
	122	-SC***C***H-
	126	-***C***H$_{(Ph)}$-, -SCCCH***C***H-
	128	-SC***C***-
	138	-***C***$^{(IV)}_{(Ph)}$-, -SCCH***C***-
	141	-S***C***-

Table S-1. ^{13}C NMR peak assignments. A table of the isotropic peaks δ_{iso} observed in P6 and P7 spectra. As no change in chemical shift was observed between the as synthesized polymers and the polymers after irradiation, they have not been separated for this table.

6. Powder X-Ray Diffraction

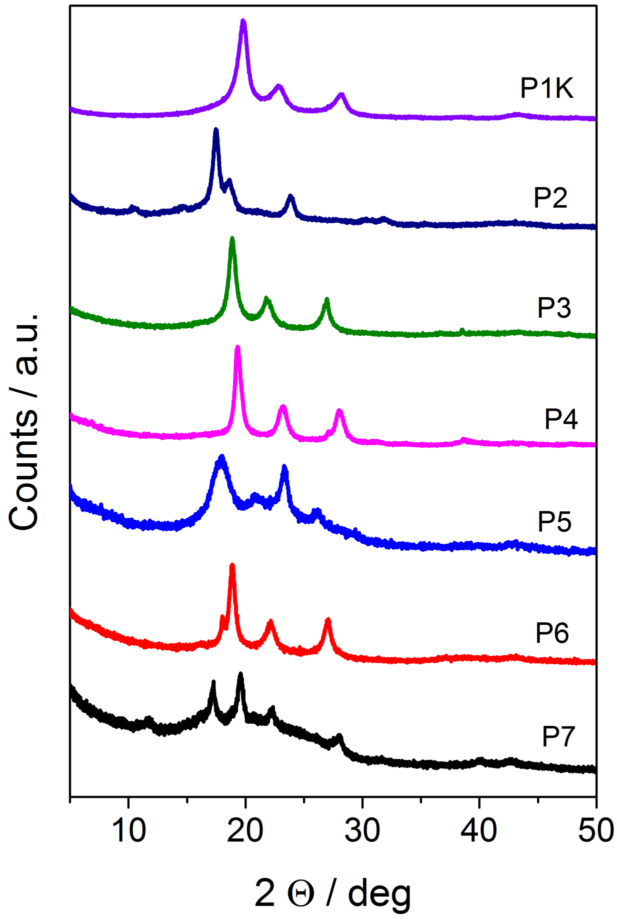

Figure S-25. PXRD patterns for **P1K**–**P7**.

Table S-2. PXRD data for **P1K** to **P7**.

**Polymer**	**2*θ* for Main Reflections / deg**	***d* / Å**
**P1K**	19.76, 22.80, 28.27	4.49, 3.90, 3.16
**P2**	17.53, 18.64, 23.78	5.06, 4.76, 3.74
**P3**	18.90, 21.79, 26.80	4.70, 4.08, 3.31
**P4**	19.20, 23.2094, 27.9688	4.62, 3.88, 3.19
**P5**	17.90, 20.97, 23.33, 26.18	4.96, 4.24, 3.81, 3.40
**P6**	18.64, 20.70, 22.02, 26.97	4.76, 4.29, 4.04, 3.31
**P7**	12.02,17.32, 19.70, 22.31, 28.02, 40.06	7.36, 5.12, 4.51, 3.99, 3.18, 2.25

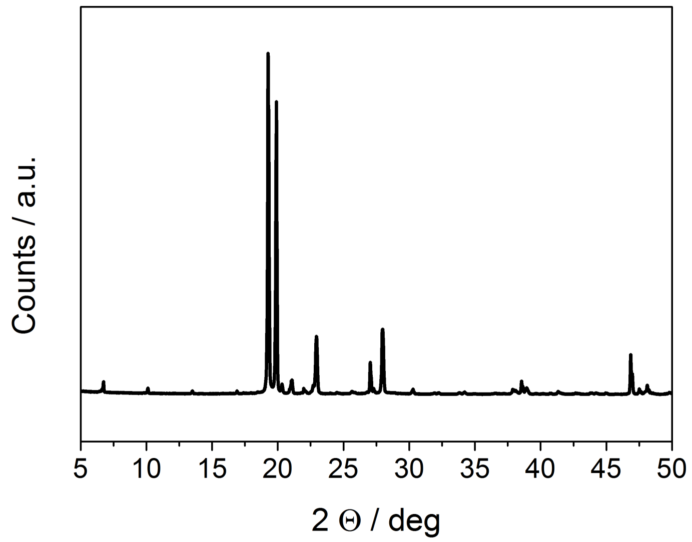

Figure S-26. PXRD pattern for **SM5** (*p*-sexiphenyl).

7. Thermogravimetric Analysis / Differential Scanning Calorimetry

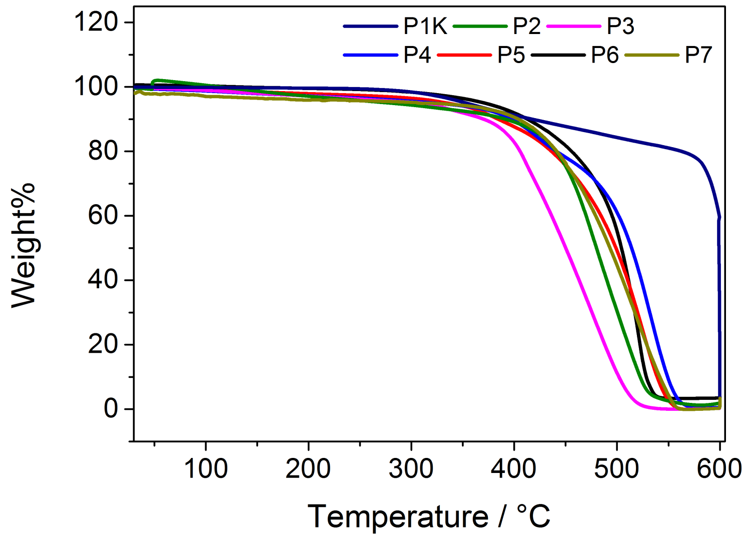

Figure S-27. TGA data for **P1K** to **P7**.

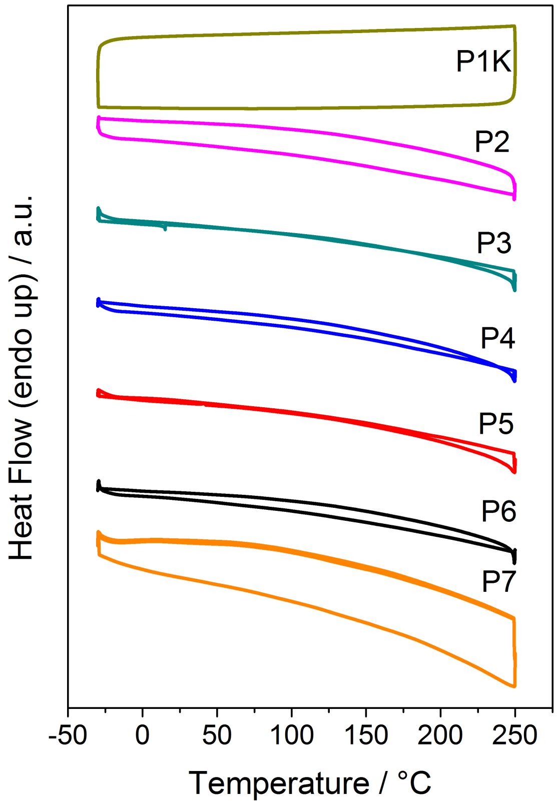

Figure S-28. DSC data for **P1K** to **P7** (the second cycle is shown).

8. Scanning Electron Microscope/Energy-dispersive X-ray spectroscopy

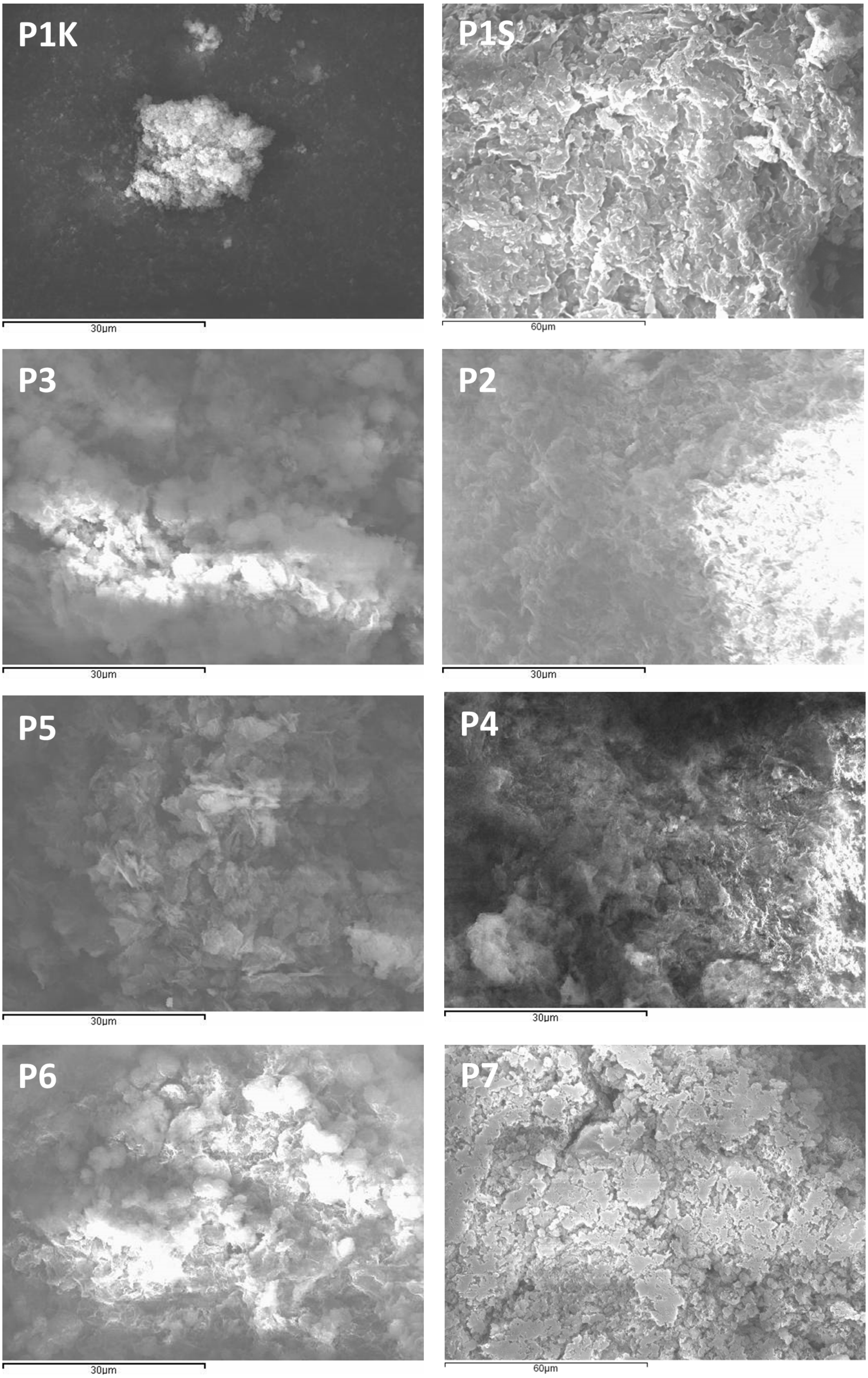

Table S-3. Energy-dispersive X-ray spectroscopy of **P1K** to **P6**.

**Polymer**	**Element / %**[a]			
	**C**	**S**	**Br**	**Pd**
**P1K**	90.15	0.15	2.29	0
**P1S**	91.05	0	1.65	0.16
**P2**	89.93	0	2.21	0.26
**P3**	93.31	0.05	1.00	0.65
**P4**	89.91	0	2.16	0.58
**P5**	89.37	0	3.66	0.93
**P6**	85.38	9.42	1.24	1.06
**P7**	70.79	11.27	2.27	0.32

[a] Average apparent composition of the sample determined *via* energy-dispersive X-ray spectroscopy in at least two points of the sample.

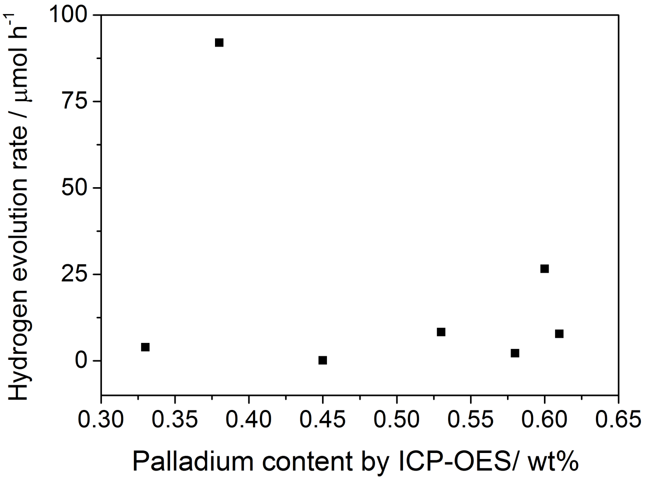

Figure S-29. Plot of Pd content in the polymer, as determined by ICP-OES, against the rate of hydrogen evolution under visible light (λ > 420 nm) for 25 mg catalyst.

9. Hydrogen Evolution Experiments for Small Molecules

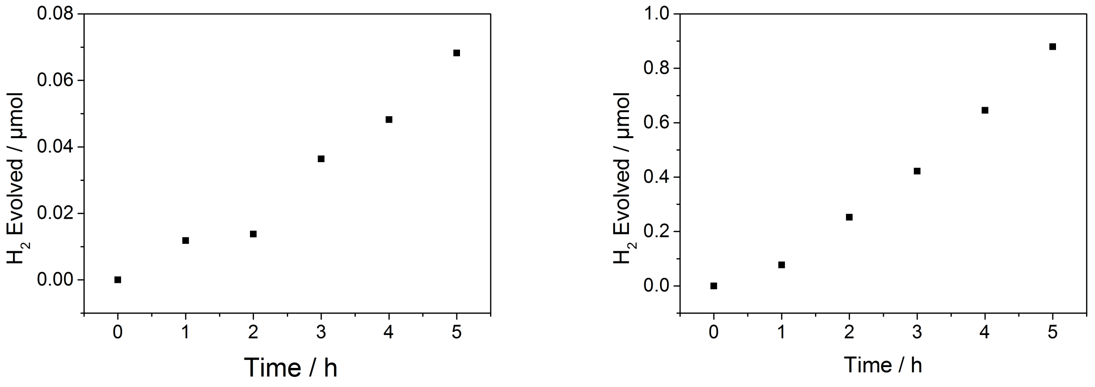

Figure S-30. Hydrogen evolution of **SM1** (25 mg) and **SM2** (25 mg) from a triethylamine/water/methanol mixture under λ > 295 nm irradiation.

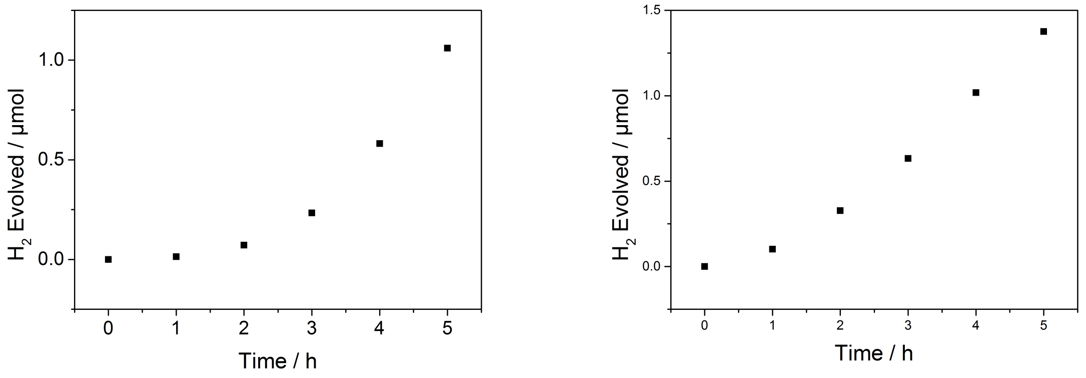

Figure S-31. Hydrogen evolution of **SM3** (25 mg) and **SM4** (25 mg) from a triethylamine/water/methanol mixture under λ > 295 nm irradiation.

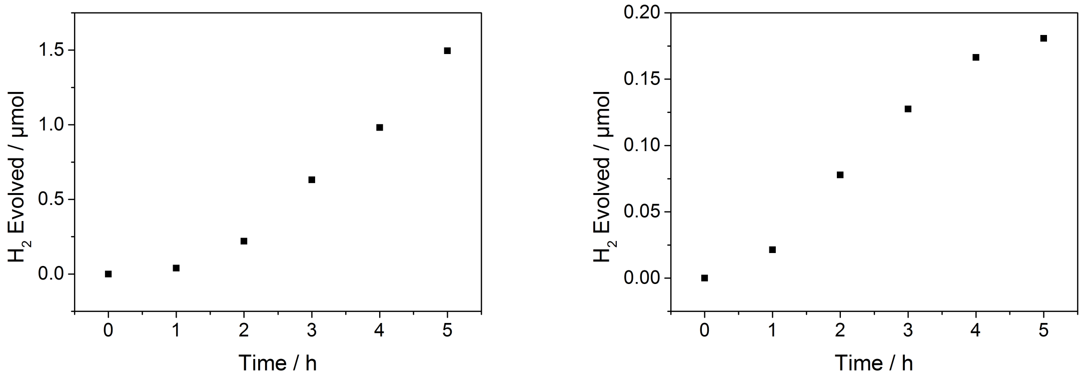

Figure S-32. Hydrogen evolution of **SM5** (25 mg) and **FSM1** (25 mg) from a triethylamine/water/methanol mixture under λ > 295 nm irradiation.

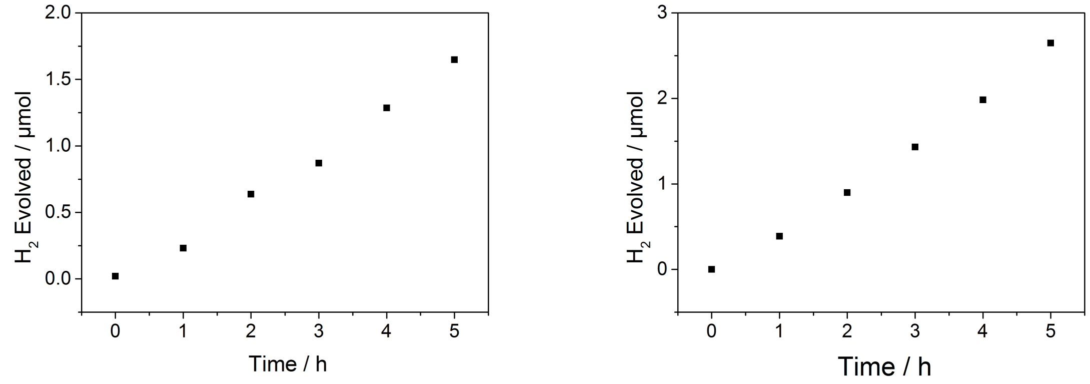

Figure S-33. Hydrogen evolution of **FSM2** (25 mg) and **FSM3** (25 mg) from a triethylamine/water/methanol mixture under λ > 295 nm irradiation.

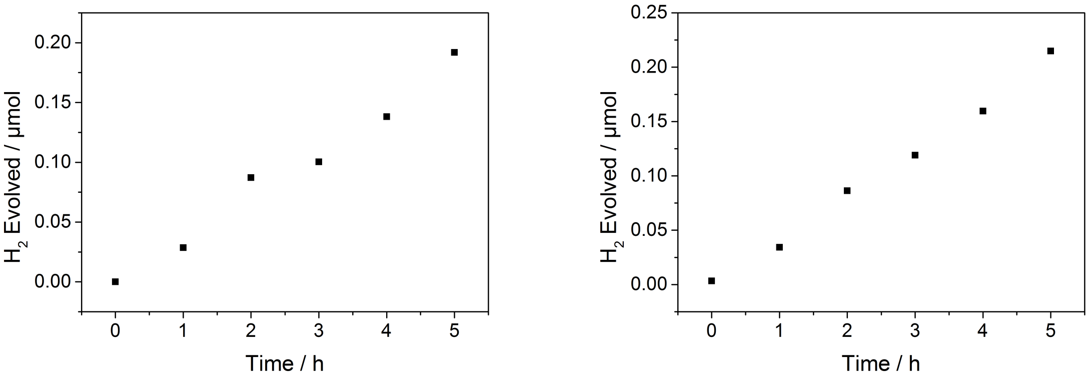

Figure S-34. Hydrogen evolution of **9*H*-carbazole** (25 mg) and **dibenzo[*b*,*d*]thiophene** (25 mg) from a triethylamine/water/methanol mixture under λ > 295 nm irradiation.

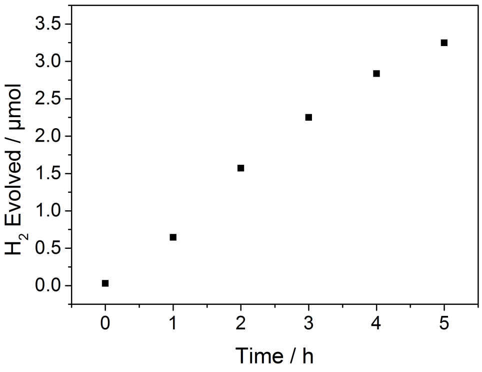

Figure S-35. Hydrogen evolution of **dibenzo[*b*,*d*]thiophene 5,5-dioxide** (25 mg) from a triethylamine/water/methanol mixture under λ > 295 nm irradiation.

Table S-4. Physical properties of the small molecules and hydrogen evolution rates.

Photocatalyst	Optical Gap [a] / eV	λ_{em} [b] / nm	λ_{max} [b] / nm	Rate of H_2 evolution > 295 nm [c] / μmol h^{-1}
**SM1**	4.41	312	250	0.013
**SM2**	3.94	342, 326	281	0.200
**SM3**	3.69	369, 353	298	0.260
**SM4**	3.55	369, 385	309	0.325
**SM5**	3.47	379, 395	313	0.368
**FSM1**	4.02	313	263, 290, 299	0.040
**FSM2**	3.76	331, 346	291, 309	0.348
**FSM3**	3.57	355, 371	308, 323	0.560
**9*H*-Carbazole**	3.63	352, 337	291, 319, 332	0.038
**Dibenzo[*b*,*d*]thiophene**	3.71	333, 346	286, 313, 325	0.040
**Dibenzo[*b*,*d*]thiophene 5,5-dioxide**	3.67	358	278, 289, 319	0.169

[a] Calculated from the UV-visible absorption on-set; [b] Measured in $CHCl_3$ solution (filtered with a 0.45 µm filter); [c] Reaction conditions: 25 mg of the polymer was suspended in 22.5 mL of a triethylamine/methanol/water solution (1:1:1 ratio), irradiated by 300 W Xe lamp (> 295 nm)

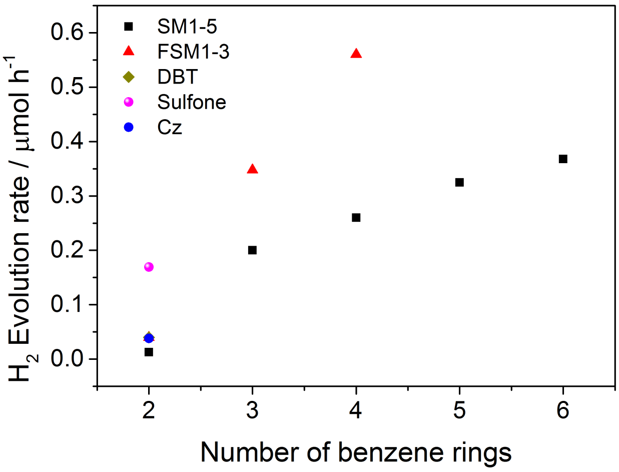

Figure S-36. Hydrogen evolution of SM1-5, FSM1-3, DBT (dibenzo[*b*,*d*]thiophene), sulfone (dibenzo[*b*,*d*]thiophene 5,5-dioxide) and Cz (9*H*-carbazole) from a triethylamine/water/methanol mixture under λ > 295 nm irradiation. Notice that the data points for FSM1, DBT, and Cz are overlapping.

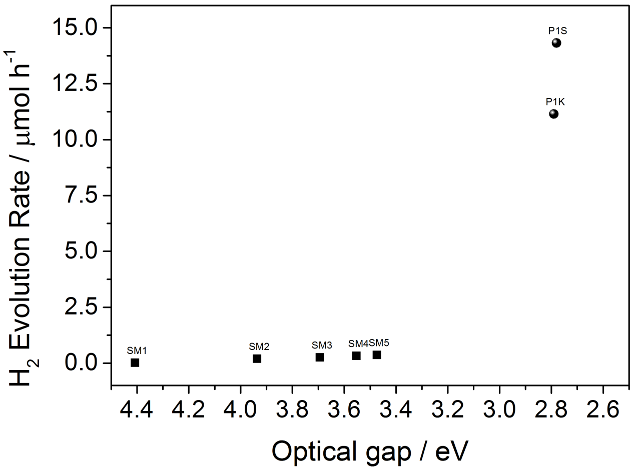

Figure S-37. Hydrogen evolution of SM1-5, P1K, and P1S from a triethylamine/water/methanol mixture under λ > 295 nm irradiation.

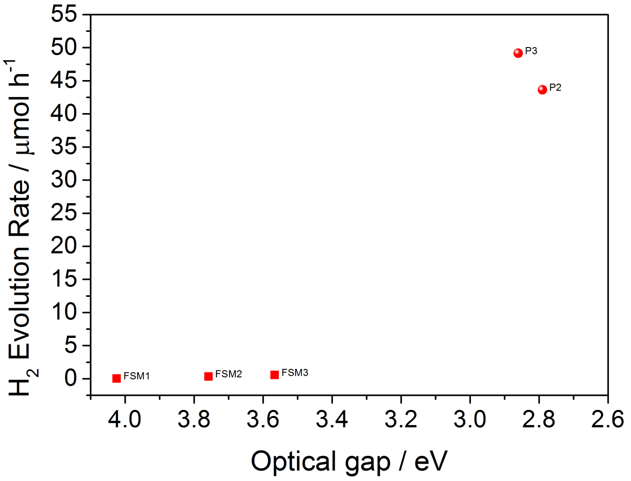

Figure S-38. Hydrogen evolution of FSM1–3, P2, and P3 from a triethylamine/water/methanol mixture under λ > 295 nm irradiation.

10. Hydrogen Evolution Experiments for Polymers

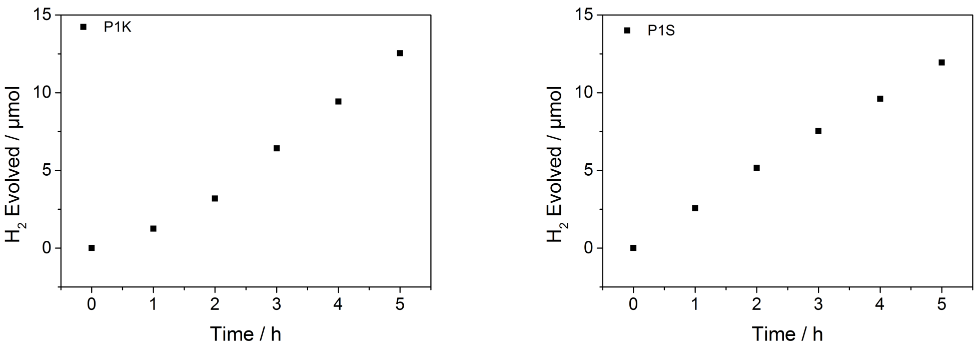

Figure S-39. H_2 evolution of **P1K** (25 mg) and **P1S** (25 mg) from a diethylamine/water mixture under λ > 295 nm irradiation.

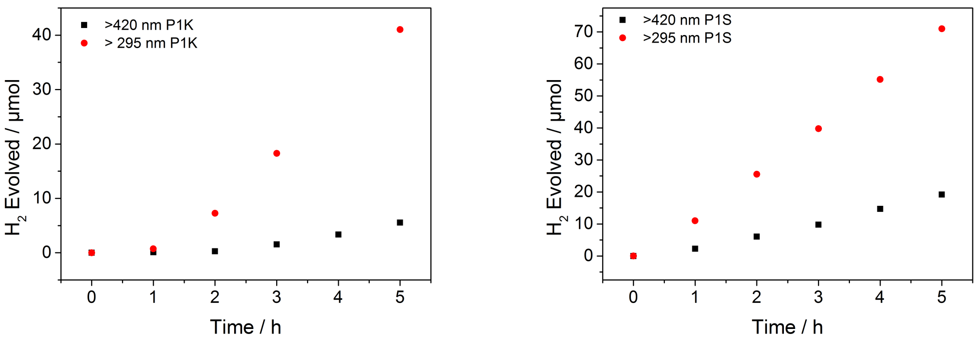

Figure S-40. H_2 evolution of **P1K** (25 mg) from a triethylamine/water/methanol mixture under λ > 295 nm and λ > 420 nm irradiation and H_2 evolution of **P1S** (25 mg) from a triethylamine/water/methanol mixture under λ > 295 nm and λ > 420 nm irradiation.

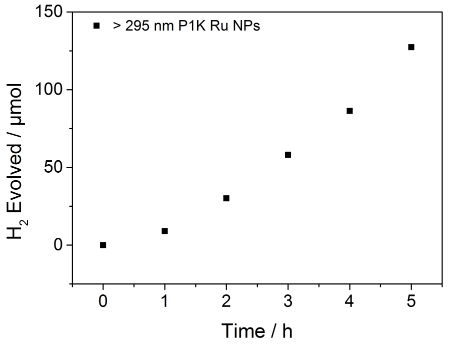

Figure S-41. H_2 evolution of **P1K** (25 mg) loaded with 3 wt.% Ru nanoparticles (from $RuCl_3 \cdot xH_2O$) from a triethylamine/water/methanol mixture under λ > 295 nm irradiation.

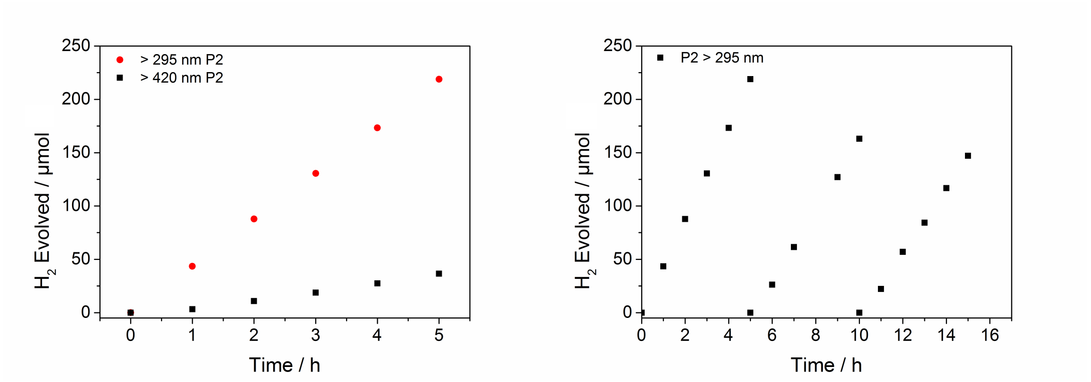

Figure S-42. H_2 evolution of **P2** (25 mg) from a triethylamine/water/methanol mixture under λ > 295 nm and λ > 420 nm irradiation and repeat runs of **P2** (25 mg) from a triethylamine/water/methanol mixture under λ > 295 nm irradiation.

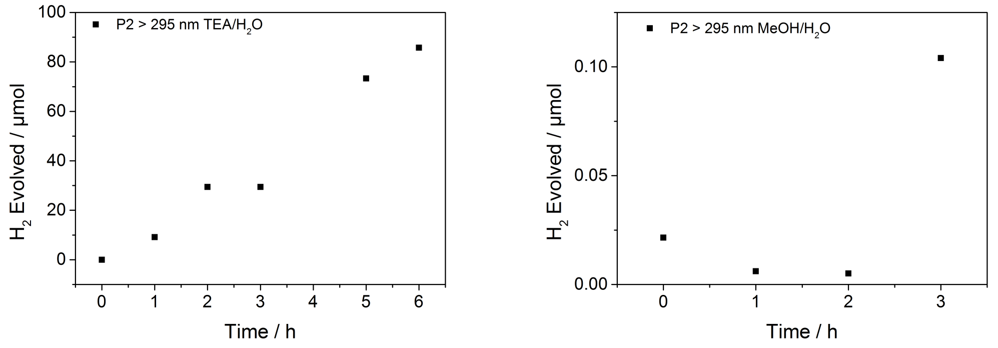

Figure S-43. H_2 evolution of **P2** (25 mg) from a triethylamine /water mixture under λ > 295 nm irradiation and **P2** (25 mg) from a methanol/water mixture under λ > 295 nm irradiation.

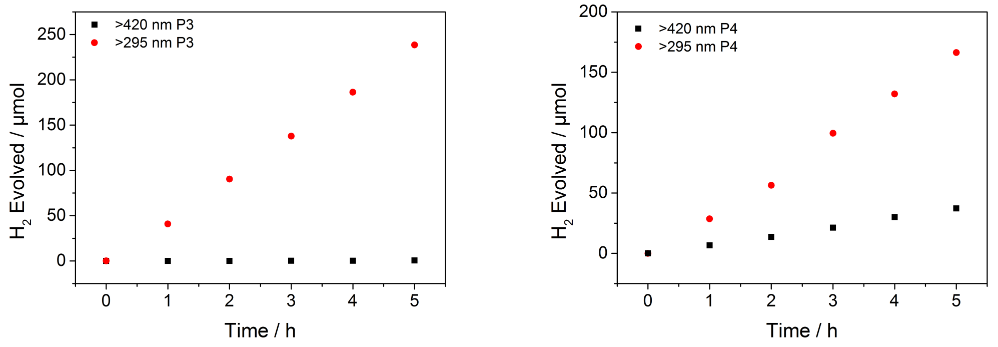

Figure S-44. H_2 evolution of **P3** (25 mg) from a triethylamine/water/methanol mixture under λ > 295 nm and λ > 420 nm irradiation and H_2 evolution of **P4** (25 mg) from a triethylamine/water/methanol mixture under λ > 295 nm and λ > 420 nm irradiation.

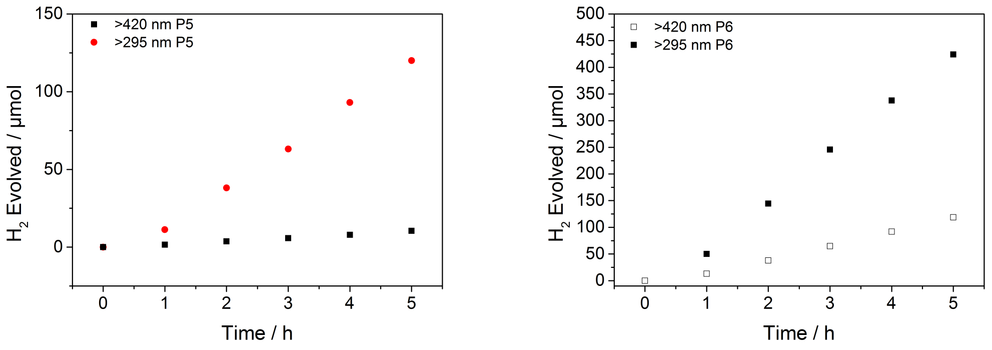

Figure S-45. H_2 evolution of **P5** (25 mg) from a triethylamine/water/methanol mixture under λ > 295 nm and λ > 420 nm irradiation and H_2 evolution of **P6** (25 mg) from a triethylamine/water/methanol mixture under λ > 295 nm and λ > 420 nm irradiation.

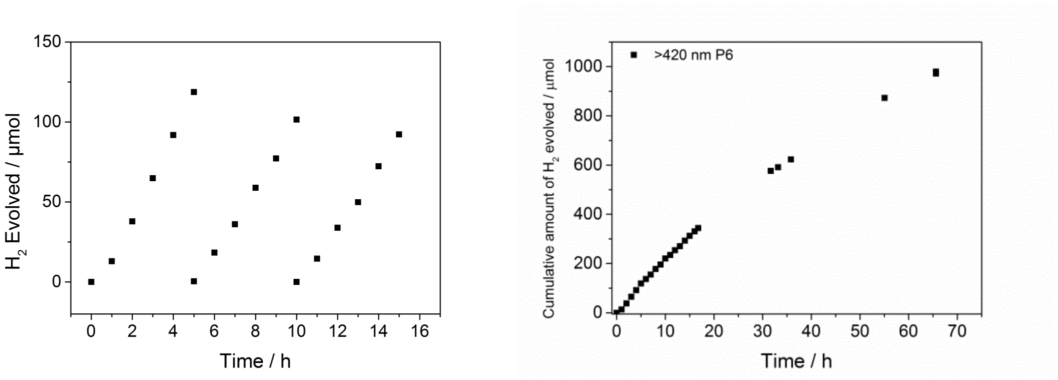

Figure S-46. Repeat H_2 evolution experiments of **P6** (25 mg) from a triethylamine/water/methanol mixture under λ > 420 nm irradiation and H_2 evolution of **P6** (25 mg) over 65 hours.

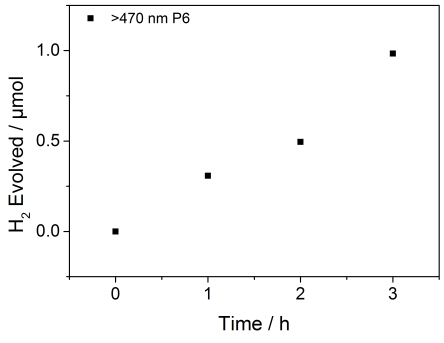

Figure S-47. H_2 evolution experiments of **P6** (25 mg) from a triethylamine/water/methanol mixture under λ > 470 nm irradiation.

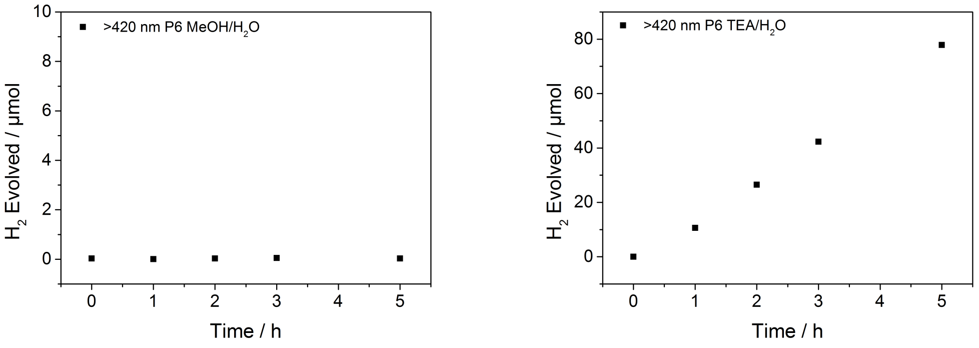

Figure S-48. H_2 evolution experiments of **P6** (25 mg) from a water/methanol mixture under λ > 420 nm irradiation and H_2 evolution of **P6** (25 mg) from a triethylamine/water mixture under λ > 420 nm irradiation.

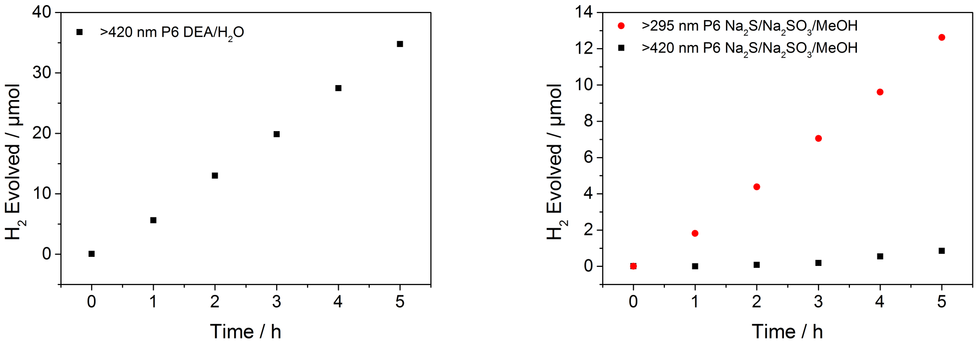

Figure S-49. H_2 evolution experiments of **P6** (25 mg) from a water/diethylamine mixture under λ > 420 nm irradiation and H_2 evolution of **P6** (25 mg) from a Na_2S (0.35 M)/Na_2SO_3 (0.25 M)/water/methanol mixture under λ > 420 nm and λ > 295 nm irradiation.

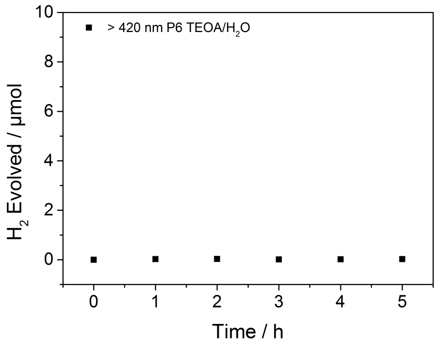

Figure S-50. H_2 evolution experiments of **P6** (25 mg) from a 10 vol.% triethanolamine/water mixture under λ > 420 nm irradiation.

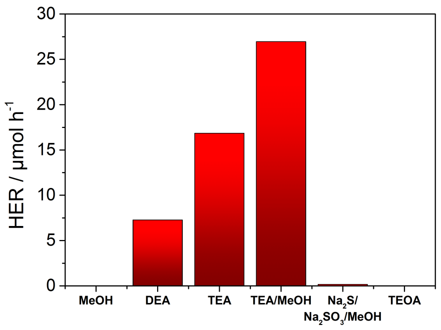

Figure S-51. Overview of H_2 evolution rates of **P6** (25 mg) using different donors under λ > 420 nm irradiation.

Table S-5. Hydrogen evolution rates for selected polymers using different scavengers and solvents.

**Entry**	**Polymer**	**Scavenger and solvent**	**Rate of H_2 evolution [a] > 295 nm / µmol h^{-1}**	**Rate of H_2 evolution [b] > 420 nm / µmol h^{-1}**
**1**	**P1K**	Diethylamine (5mL) / water (20 mL)	2.4	ND
**2**	**P1S**	Diethylamine (5mL) / water (20 mL)	2.4	ND
**3[c]**	**P1K**	Triethylamine (7.5 mL) / MeOH (7.5 mL) / water (7.5 mL)	25.6	ND
**4**	**P2**	Triethylamine (12 mL) / water (12 mL)	14.7	ND
**5**	**P2**	MeOH (7.5 mL) / water (7.5 mL)	0	ND
**6**	**P6**	Diethylamine (5 mL) / water (20 mL)	ND	7.3
**7**	**P6**	Triethylamine (1 mL) / water (20 mL)	ND	16.8
**8**	**P6**	MeOH (5 mL) / water (20 mL)	ND	0
**9**	**P6**	Na_2S (0.35 M) / Na_2SO_3 (0.25 M) / water (25 mL) / MeOH (5 mL)	2.7	0.2
**10**	**P6**	10 vol.% Triethanolamine/water	0	ND

[c] Loaded with 3 wt.% Ru nanoparticles from $RuCl_3$.

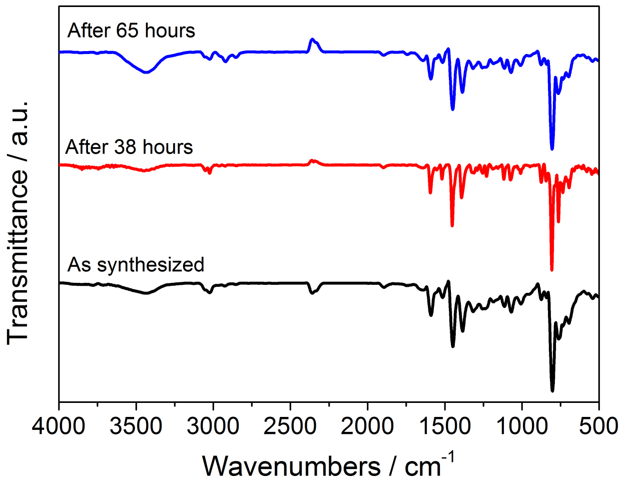

Figure S-52. FT-IR spectra of **P6** before and after irradiation with visible light (λ > 420 nm) for 38 hours and 65 hours in a triethylamine/water/methanol mixture.

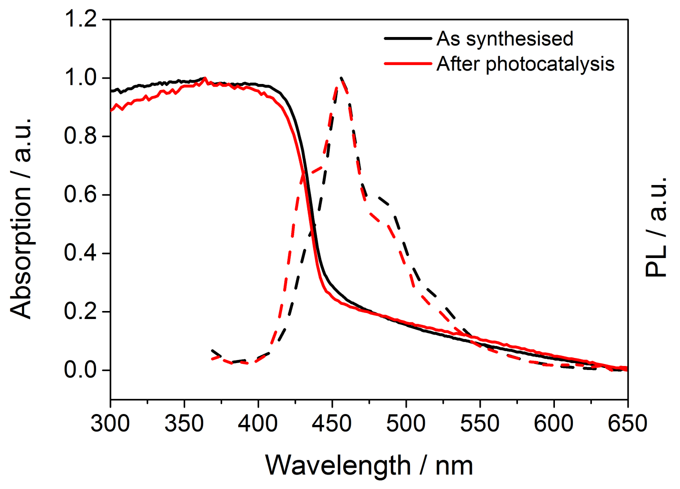

Figure S-53. UV/Vis and PL spectra (λ_{exc} = 360 nm) of **P6** before and after irradiation with visible light (λ > 420 nm) for 65 hours in a triethylamine/water/methanol mixture.

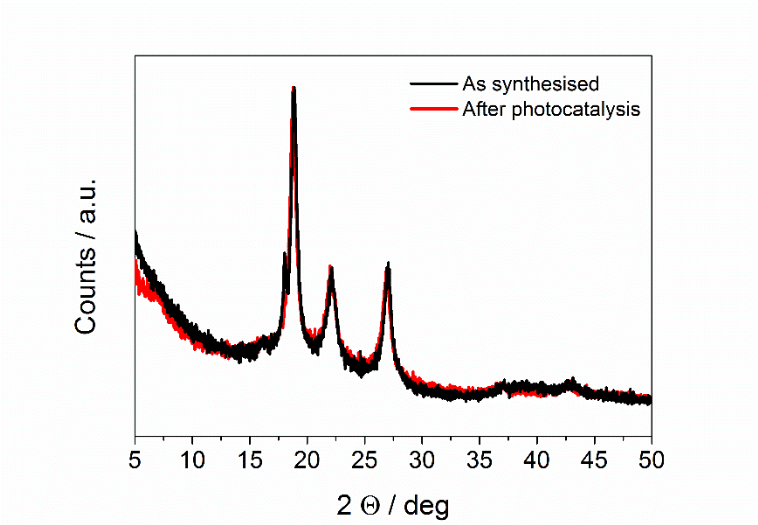

Figure S-54. PXRD spectra of **P6** before and after irradiation with visible light (λ > 420 nm) for 65 hours in a triethylamine/water/methanol mixture.

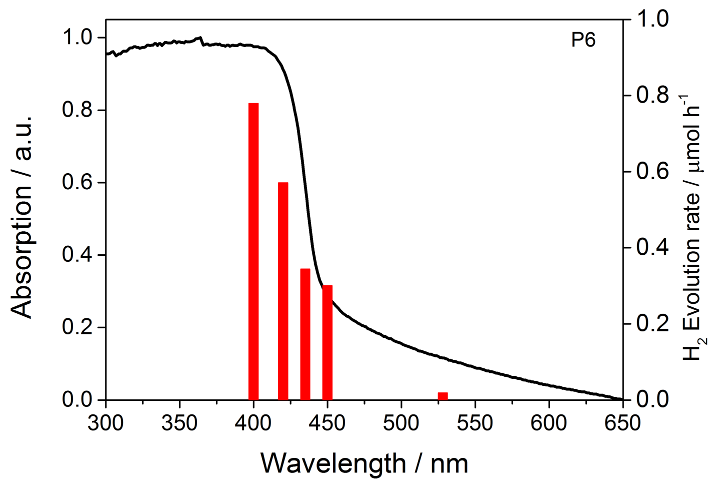

Figure S-55. Wavelength-dependency of the H_2 evolution of **P6** (40 mg) from a triethylamine/water/methanol mixture.

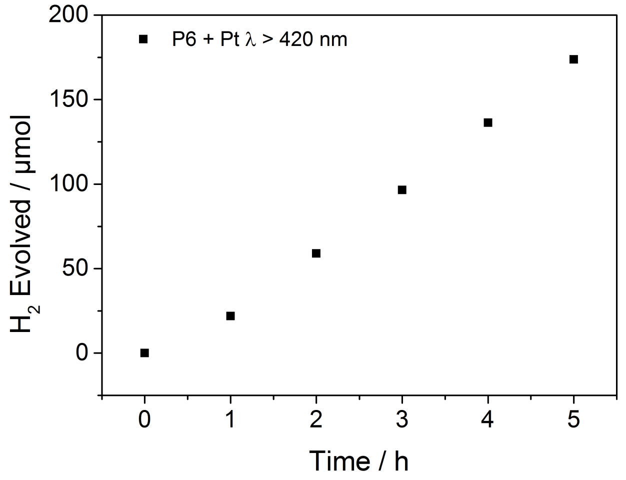

Figure S-56. H_2 evolution experiments of **P6** (25 mg) from a triethylamine/water/methanol mixture under λ > 420 nm irradiation loaded with Pt nanoparticles (> 100 nm). Pt nanoparticles were formed *via* citric acid reduction of H_2PtCl_6 (95 °C, 4 h) and dialyzed for 5 days. HER (**P6** + Pt NPs, λ > 420 nm) = 38.1 µmol h^{-1}.

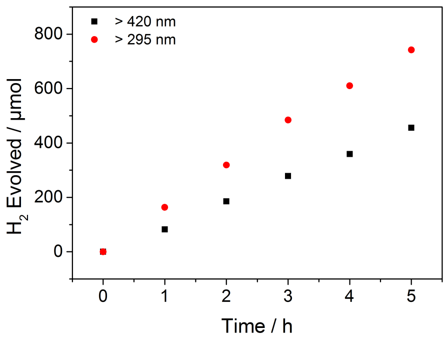

Figure S-57. H_2 evolution of **P7** (25 mg) from a triethylamine/water/methanol mixture under λ > 295 nm and λ > 420 nm irradiation.

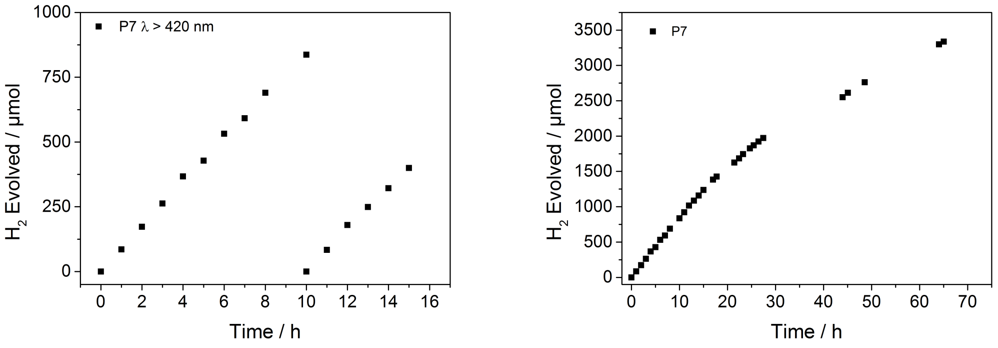

Figure S-58. Repeat H_2 evolution experiments of **P7** (25 mg) from a triethylamine/water/methanol mixture under λ > 420 nm irradiation and H_2 evolution of **P7** (25 mg) over 65 hours.

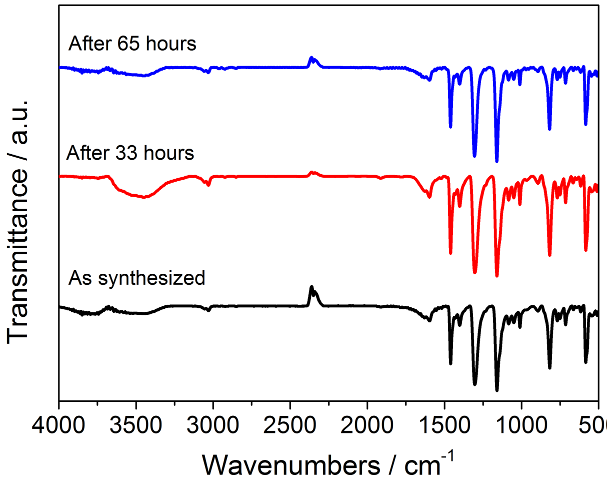

Figure S-59. FT-IR spectra of **P7** before and after irradiation with visible light (λ > 420 nm) for 33 hours and 65 hours in a triethylamine/water/methanol mixture.

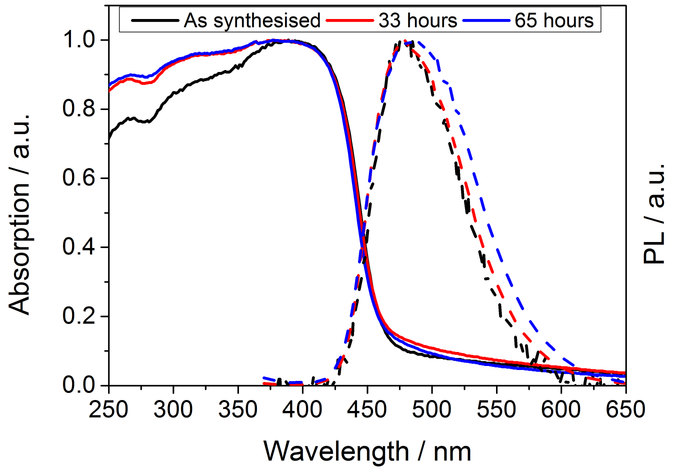

Figure S-60. UV/Vis and PL spectra (λ_{exc} = 360 nm) of **P7** before and after irradiation with visible light (λ > 420 nm) for 33 hours and 65 hours in a triethylamine/water/methanol mixture.

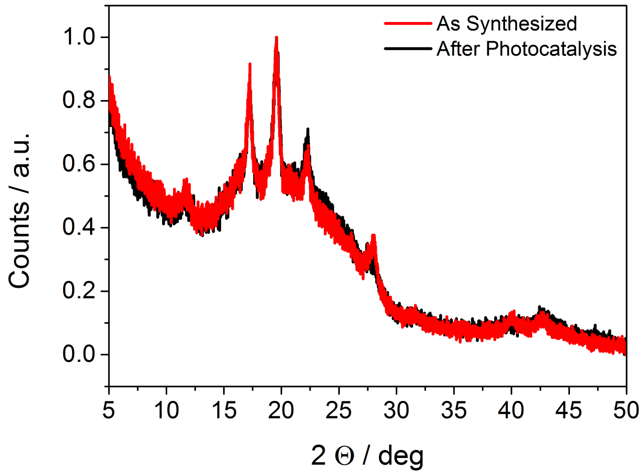

Figure S-61. PXRD spectra of **P7** before and after irradiation with visible light (λ > 420 nm) for 65 hours in a triethylamine/water/methanol mixture.

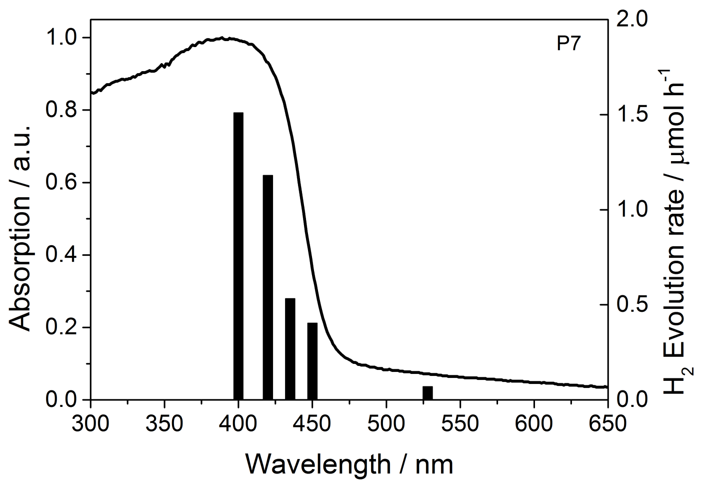

Figure S-62. Wavelength-dependency of the H_2 evolution of **P7** (40 mg) from a triethylamine/water/methanol mixture.

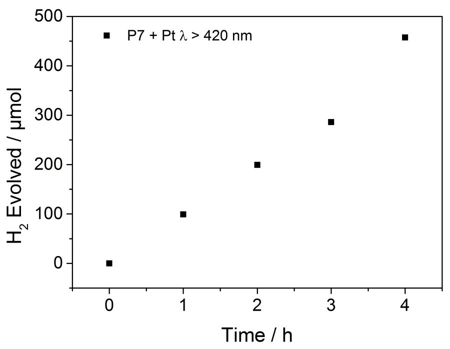

Figure S-63. H_2 evolution experiments of **P7** (25 mg) from a triethylamine/water/methanol mixture under λ > 420 nm irradiation loaded with Pt nanoparticles (> 100 nm). Pt nanoparticles were formed *via* citric acid reduction of H_2PtCl_6 (95 °C, 4 h) and dialyzed for 5 days. HER (**P6** + Pt NPs, λ > 420 nm) = 116.1 µmol h^{-1}.

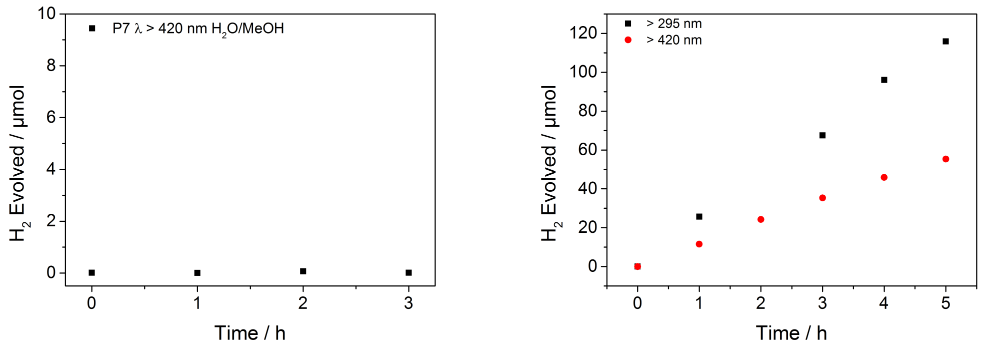

Figure S-64. H_2 evolution of **P7** (25 mg) from a water (20 mL)/methanol (5 mL) mixture under λ > 420 nm irradiation (left) and from a Na_2S (0.35 M)/Na_2SO_3 (0.25 M)/water/methanol mixture under λ > 420 nm and λ > 295 nm irradiation (right).

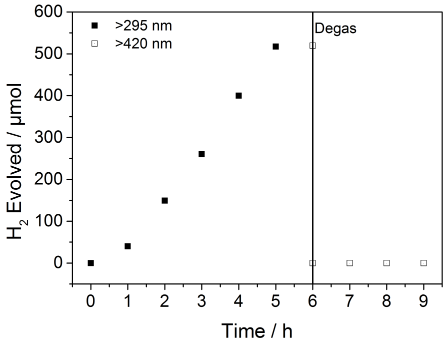

Figure S-65. H_2 evolution experiment of commercial rutile (25 mg) in 20 vol. % methanol in water loaded with 1 wt.% Pt (from H_2PtCl_6) under λ > 295 nm irradiation (for 5 hours). A rate of 108 µmol h^{-1} was observed. By contrast no activity was found under visible light irradiation (λ > 420 nm).

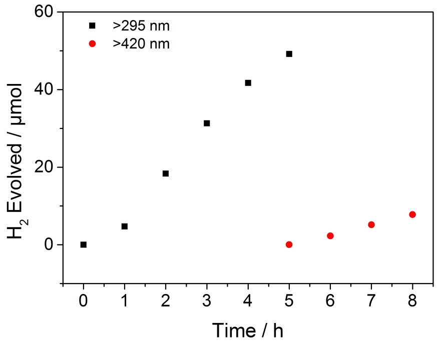

Figure S-66. H_2 evolution experiment of commercial g-C_3N_4 (25 mg) in 10 vol% triethanolamine in water loaded with 3 wt.% Pt (from H_2PtCl_6) under λ >295 nm irradiation (for 5 hours). A rate of 11.2 µmol h^{-1} was observed. After degassing and illumination with visible light irradiation (λ > 420 nm) a rate of 2.7 µmol h^{-1} was observed.

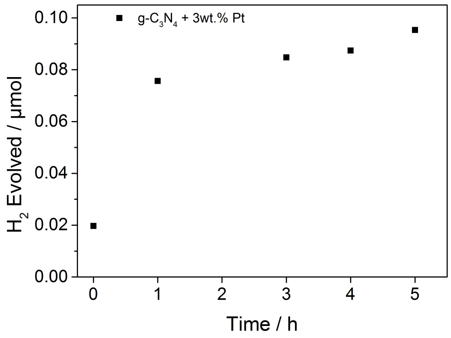

Figure S-67. H_2 evolution experiments of commercial g-C_3N_4 (25 mg) from a triethylamine/water/methanol mixture water loaded with 3 wt.% Pt (from H_2PtCl_6) under λ > 420 nm irradiation.

11. Apparent Quantum Yields

For determining the apparent quantum yield, the photocatalyst (P1K, P6, P7 and CP-CMP10; 40 mg) was suspended in water (12 mL), methanol (12 mL) and TEA (12 mL). In the case of g-C_3N_4, the photocatalyst was suspended in water (35 mL) and triethanolamine (5 mL) and loaded with Pt nanoparticles (3 wt.% from H_2PtCl_6 solution). Hydrogen evolution was studied using a focused λ = 420 nm LED (67.7 mW cm^{-2}) controlled by an IsoTech IPS303DD Power Supply. An area of 5.73 cm^2 was illuminated and the light intensity was measured with a ThorLabs S120VC photodiode power sensor controlled by a ThorLabs PM100D Power and Energy Meter Console. The apparent quantum yields were estimated using Equation (1):

$$\Phi_{H_2} = 2\times\frac{moles\ of\ hydrogen\ evolved}{moles\ of\ the\ incident\ photons} \quad \text{Equation (1)}$$

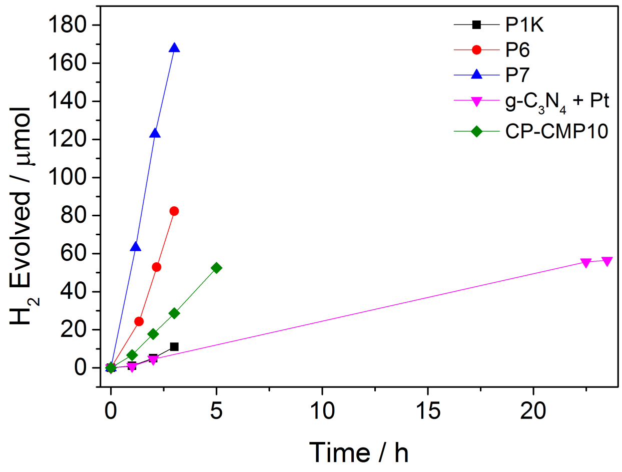

Figure S-68. H_2 evolution experiments under λ = 420 nm illumination using a LED.

Table S-6. Calculated apparent quantum yields.

Photocatalyst	Calculated apparent quantum yield
**P1K**	0.13%
**P6**	1.10%
**P7**	2.25%
**CP-CMP10**	0.42%
**g-C_3N_4**	0.10%

12. TD-DFT Calculations

Absorption on-set

We calculated the lowest vertical singlet-singlet excitation energy (LVEE), for conjugated *para*-phenylene oligomers (SM1–SM10) and their planarized 9*H*-fluorene-type analogs (FSM1-FSM11, see Fig. S-68 for the used nomenclature for the planarized 9*H*-fluorene-type analog structure longer than discussed in the main paper, as well as 9*H*-carbazole and dibenzo[*b*,*d*]thiophene versions) using Time-Dependent Density Functional Theory (TD-DFT). For the small oligomers, one would expect that this LVEE would coincide with the maximum of the first absorption peak and that all experimental absorption intensity at lower-energy/longer wavelength, *i.e.*, between the experimental on-set of light absorption (the optical gap) and first peak maximum would be due to vibrational broadening. For the polymers and longer oligomers, the match might be more complicated due to inhomogeneous broadening and scattering.

For each oligomer we considered only the lowest energy conformer, which was found using the MacroModel (version 9.9, Schrödinger, LLC, New York, NY, 2011) conformer-searching tool. The procedure uses a low-mode (LMOD) sampling approach,[6] which follows the low frequency eigenvectors of the structure. We used 10,000 search steps with maximum and minimum move distances of 3 and 20 Å, respectively. All structures within an energy window of 200 kJ mol^{-1} of the lowest energy conformation were retained. This number of steps was chosen as repeats of the calculations found no new low energy structures, with each conformation sampled multiple times within an individual run. For geometry optimization, the convergence criterion was a remaining RMS force of 0.05 kJ mol^{-1} $Å^{-1}$.

The lowest energy conformer for each of the oligomers was subsequently optimized using ground state density functional theory (DFT) and their vertical absorption spectra calculated using TD-DFT. All DFT and TD-DFT calculations employed the B3LYP[7-10] (XC-)functional, the DZP basis-set,[11] and were run using the Turbomole 6.5 code[12-14] and the NWChem 6.5 code.[15] Calculations were performed for two scenarios; (i) gas phase, and (ii) a chloroform solution. The latter calculations employed the COSMO solvation model with a dielectric permittivity of 4.81.

The calculated LVEE values in the gas phase and chloroform are shown in Fig. S-69 and S-70 and show a similar behavior as the experimental optical gaps in the main paper. For short oligomer lengths, the oligo(*p*-phenylene)s have consistently higher LVEE values than their 9*H*-fluorene-based counterparts (as well as than the 9*H*-carbazole and dibenzo[*b*,*d*]thiophene; oligomers not studied experimentally). In contrast, in the long oligomer limit all materials have very similar LVEE values, in line with the very similar optical gaps for all polymers. The origin of subtle differences between our predictions and experiment; for example, the fact that our calculations suggest that P1 should have

a larger optical gap than P3, in contrast to experiment, might be due to the materials having different degrees of polymerization (molar-mass dispersity), as well as slight deviations in the quality of description of the different materials by TD-B3LYP.

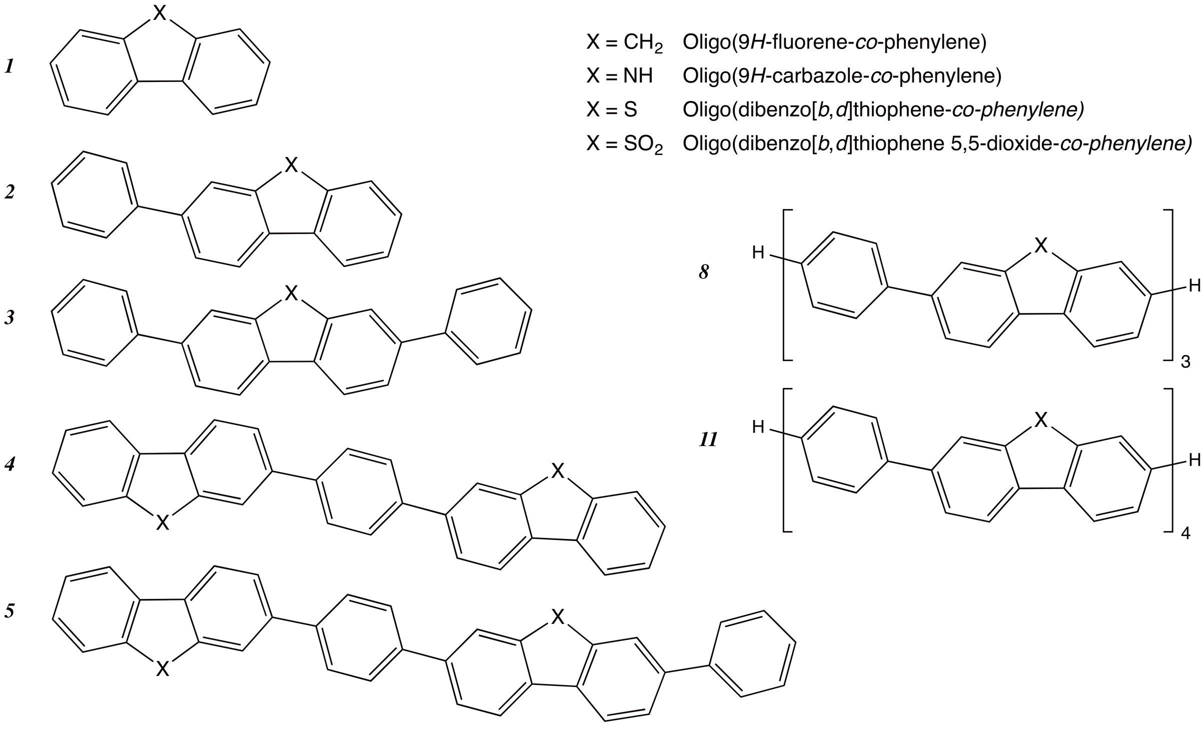

Figure S-69. Structures of the planarized 9*H*-fluorene-type oligomers studied.

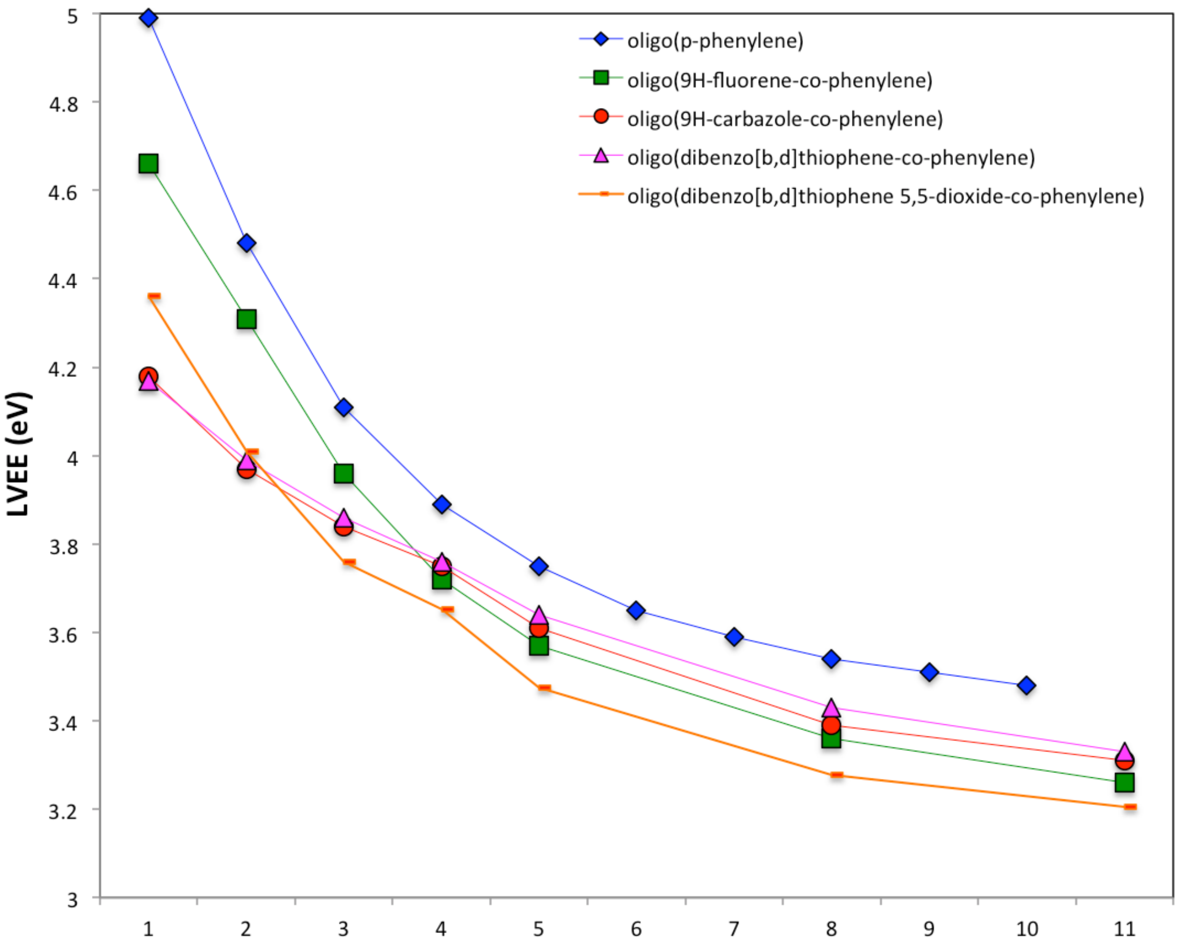

Figure S-70. TD-B3LYP predicted LVEE values of the different oligomers in the gas phase. The numbers along the x-axis correspond to the number of phenylene moieties in the oligomer minus one, in line with the nomenclature used in the main paper.

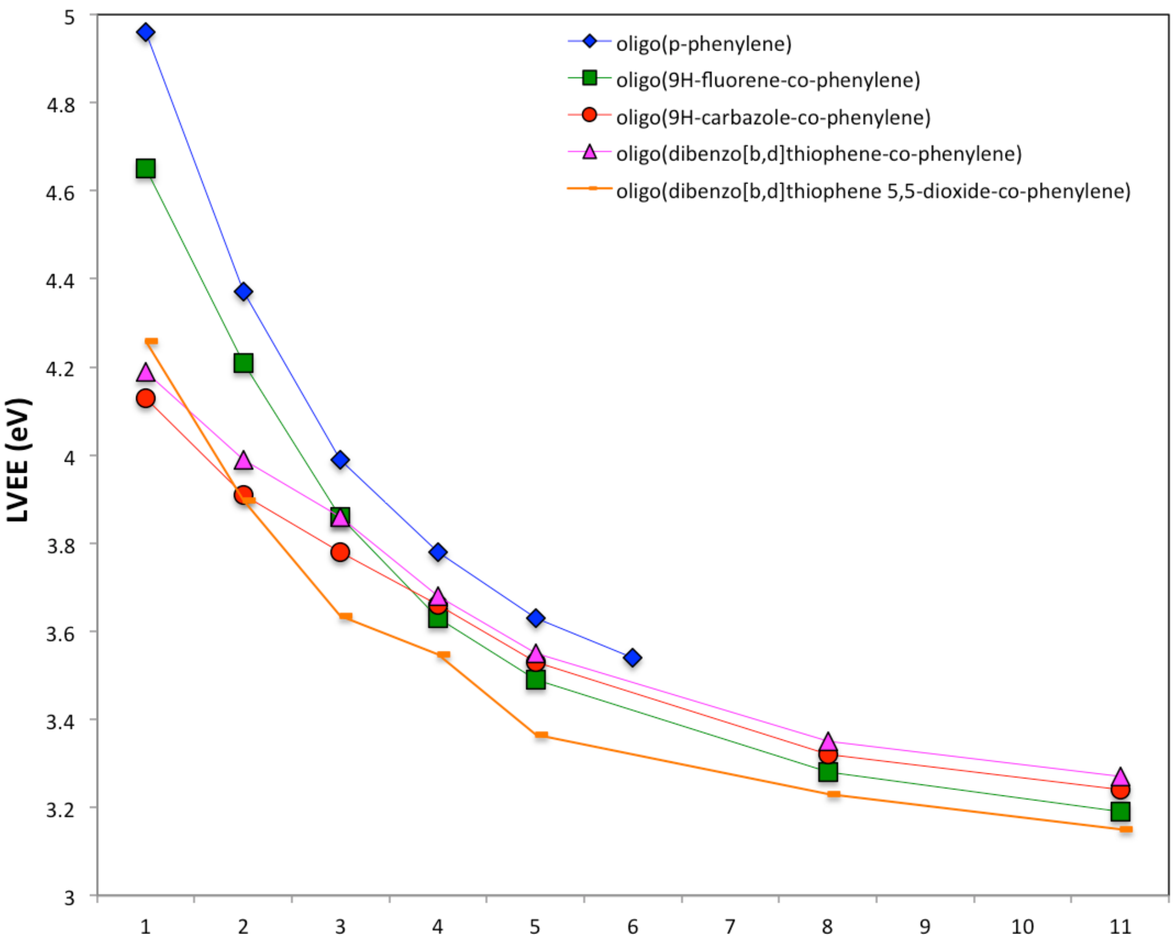

Figure S-71. TD-B3LYP predicted LVEE values of the different oligomers in chloroform. The numbers along the x-axis correspond to the number of phenylene moieties in the oligomer minus one, in line with the nomenclature used in the main paper.

Thermodynamic ability of polymers to drive water splitting half-reactions

For the different oligomers to be able to act as overall water splitting photocatalysts, they need to be able to thermodynamically drive both the reduction of protons and the oxidation of water. Following our previous work on poly(*p*-phenylene)[16] and carbon nitride,[17] we calculated for the different oligomers the standard reduction potentials of half-reactions in which the exciton, formed upon absorption of light, donates either an electron or a hole (half reactions **1** and **2**). We also calculated similar standard reduction potentials for free electrons and holes that can be formed through dissociation of the exciton (half reactions **3** and **4**). The relevant half-reactions, written following convention in the form of reductions, are:

$$P^{+} + e^{-} \rightleftarrows P^{*} \qquad (1)$$

$$P^{*} + e^{-} \rightleftarrows P^{-} \qquad (2)$$

$$P + e^- \rightleftarrows P^- \quad (3)$$

$$P^+ + e^- \rightleftarrows P \quad (4)$$

Here P stands for the neutral oligomer in its electronic ground state, P^* the oligomer in its lowest electronically excited state, and P^+ and P^- for the oligomer with a free hole and free electron respectively. In half reactions **1** and **3**, the polymer donates an electron, *i.e.* act as a reductant, and the reaction in practice will run in the opposite direction. The potentials for the oligomer half-reactions are than compared with the potentials of the proton reduction and water oxidation half-reactions:

$$2H^+ + 2e^- \rightleftarrows H_2 \quad (5)$$

$$O_2 + 4H^+ + 4e^- \rightleftarrows 2H_2O \quad (6)$$

Again written as reductions. When the standard reduction potentials of half-reactions **1** (IP^*) and/or **3** (EA) are more negative than that of half-reaction **5**, proton reduction is thermodynamically feasible. Similarly, when the standard reduction potentials of half-reactions **2** (EA^*) and/or **4** (IP) are more positive than that of half-reaction **6**, water oxidation is thermodynamically feasible.

We also considered the half-reaction corresponding to the 2-electron oxidation of triethylamine, the sacrificial hole-acceptor used experimentally, which, when written as a reduction, is:

$$NH(CH_2CH_3)_2 + CH_3CHO + 2H^+ + 2e^- \rightarrow NH(CH_2CH_3)_3 + H_2O \quad (7)$$

Oxidation of triethylamine is thermodynamically feasible when the standard reduction potentials of half-reactions **2** and/or **4** are more positive than that of half-reaction **7**.

The standard reduction potentials for half-reactions **1-7** were calculated using a similar set-up as the absorption on-set calculations (see above) with one important difference. We perform our calculations using the relative dielectric permittivity of water (80.1) rather than the chloroform value in the COSMO solvation model. Use of the dielectric permittivity of methanol or triethylamine instead would only make a small difference as shown in our previous work on poly(*p*-phenylene).[16] The potentials for the half-reactions **5-7** by necessity include nuclear relaxation and even a vibrational free-energy contribution. As found in our previous work,[16,17] the magnitude of the contribution of vibrational free-energy to the potentials of half-reactions **1-4** is small, because of the structural similarity between the compounds on either side of the reaction arrow, and large for half-reactions **5-7**. Finally, just as in our previous work,[16,17] for computational reasons we used for the potential of half-reaction **5**, the experimentally measured absolute potential of the standard hydrogen electrode.[18]

Fig. S-71 to S-75 show the (TD-)B3LYP predicted potentials for SM1-7, FSM1-11, the 9*H*-carbazole version of FSM1-11, the dibenzo[*b*,*d*]thiophene version of FSM1-11, and the dibenzo[b.d]thiophene

5,5-dioxide version of FSM1-11 respectively. Clearly in all cases the IP* and EA potentials are predicted to be considerably more negative than the proton reduction potential and hence all oligomers should be able to reduce protons to hydrogen. In contrast, water oxidation is predicted to be generally endothermic, where for most oligomers studied, the EA* and IP potentials are predicted to lie below (*i.e.*, be more negative than) the water oxidation potential. Finally, in line with the successful use of triethylamine as sacrificial electron donor, triethylamine oxidation is overall predicted to be exothermic, where the calculated EA* and IP potentials are considerably more positive than the triethylamine oxidation potential (the same also holds for methanol oxidation, not shown, though there the overpotential is smaller as the methanol oxidation reaction is predicted to have a potential of +0.29 V vs. +0.03 V in the case of triethylamine).

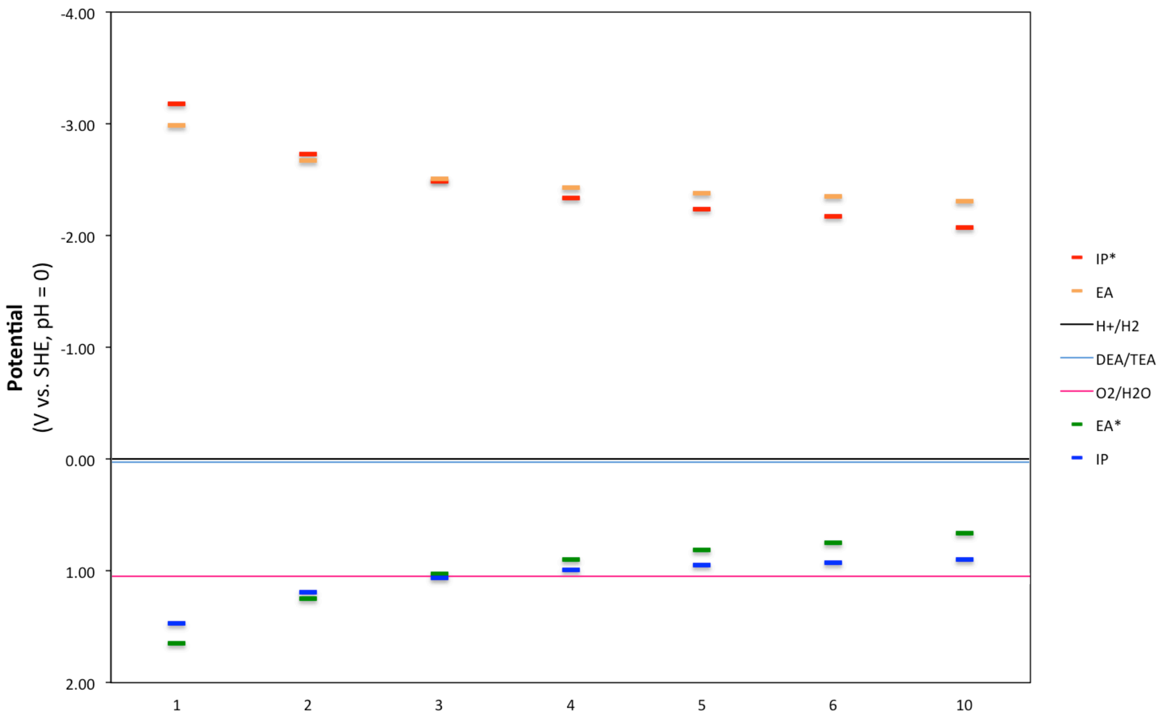

Figure S-72. (TD-)B3LYP predicted potentials for the oligo(*p*-phenylene)s *vs* the standard reduction potentials of proton reduction, water oxidation and triethylamine oxidation (all potentials calculated at pH = 0, the numbers along the x-axis correspond to the number of phenylene moieties in the oligomer minus one, in line with the nomenclature used in the main paper).

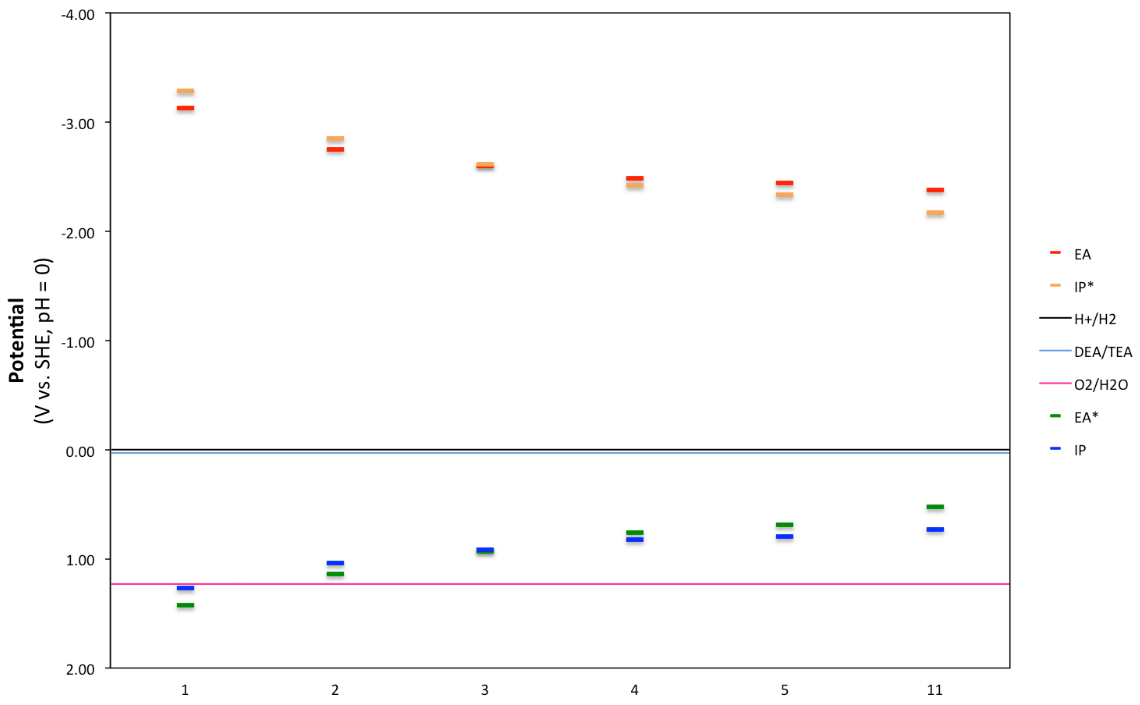

Figure S-73. (TD-)B3LYP predicted potentials for the oligo(9*H*-fluorene-*co*-phenylene)s *vs* the standard reduction potentials of proton reduction, water oxidation and triethylamine oxidation (all potentials calculated at pH = 0, the numbers along the x-axis correspond to the number of phenylene moieties in the oligomer minus one, in line with the nomenclature used in the main paper).

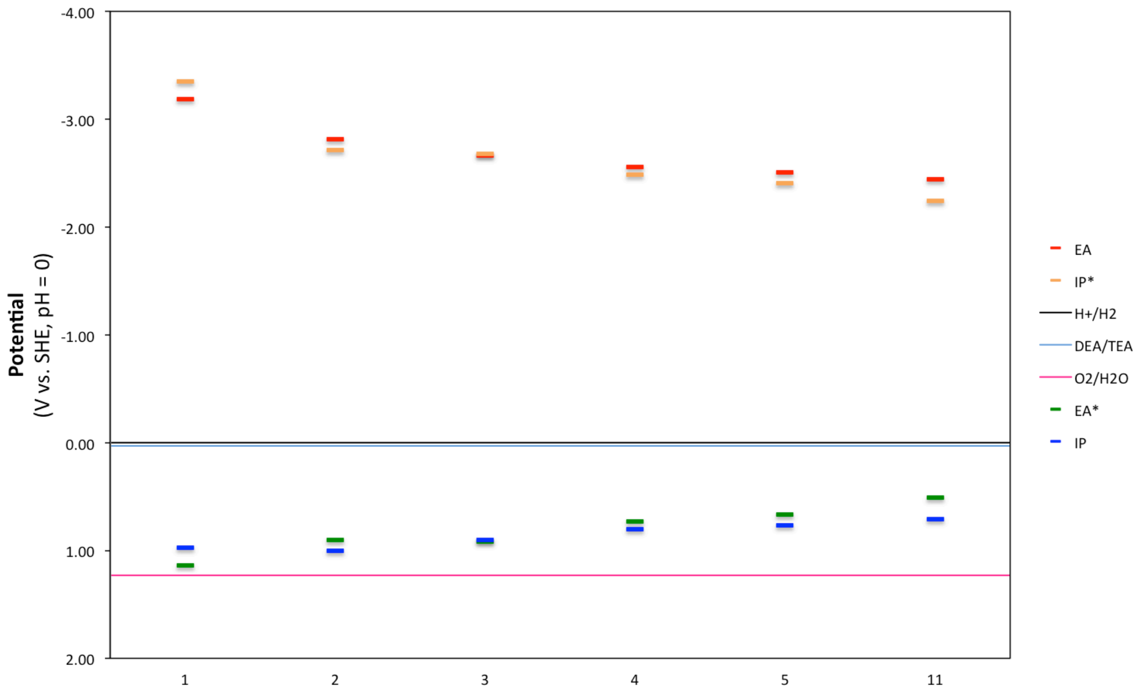

Figure S-74. (TD-)B3LYP predicted potentials for oligo(9*H*-carbazole-co-phenylene)s *vs* the standard reduction potentials of proton reduction, water oxidation and triethylamine oxidation (all potentials calculated at pH = 0, the numbers along the x-axis correspond to the number of phenylene moieties in the oligomer minus one, in line with the nomenclature used in the main paper).

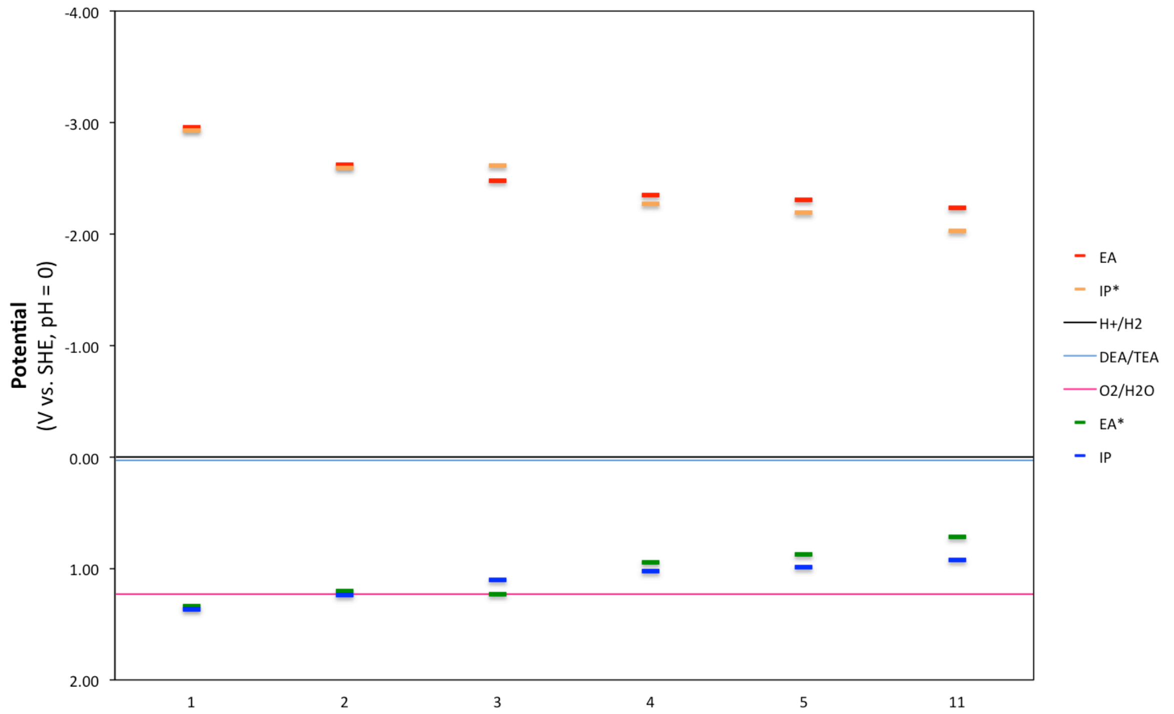

Figure S-75. (TD-)B3LYP predicted potentials for oligo(dibenzo[*b*,*d*]thiophene-co-phenylene)s *vs* the standard reduction potentials of proton reduction, water oxidation and triethylamine oxidation (all potentials calculated at pH = 0, the numbers along the x-axis correspond to the number of phenylene moieties in the oligomer minus one, in line with the nomenclature used in the main paper).

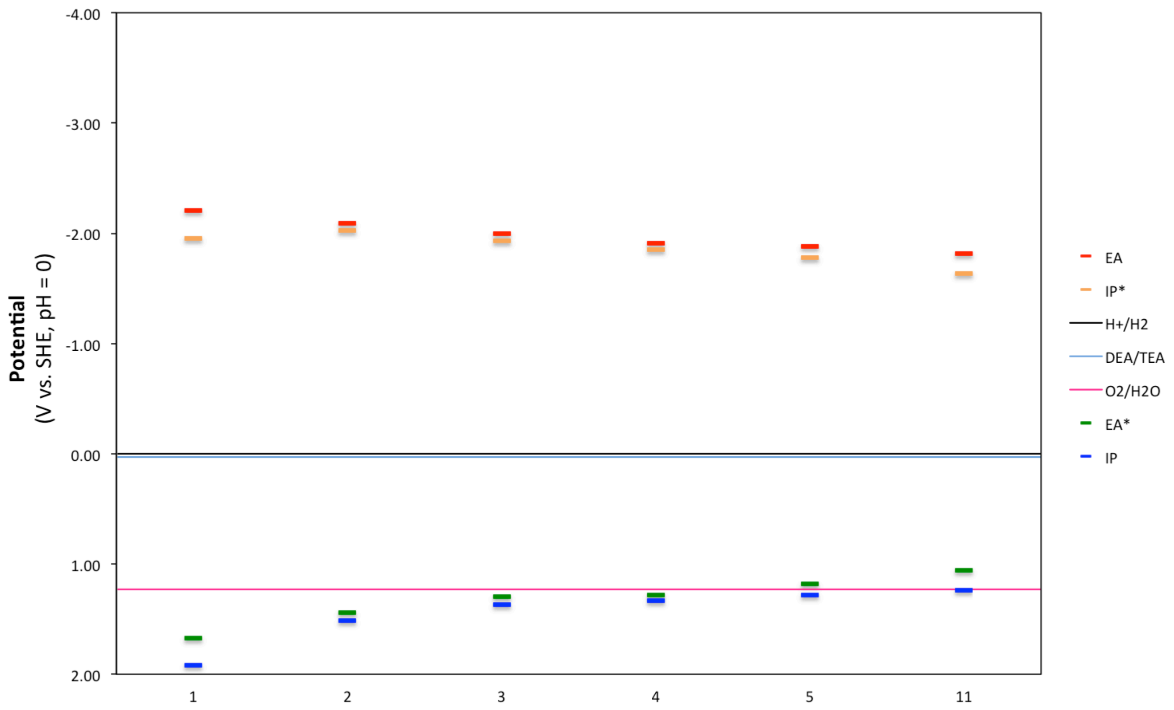

Figure S-76. (TD-)B3LYP predicted potentials for oligo(dibenzo[*b*,*d*]thiophene 5,5-dioxide-co-phenylene)s *vs* the standard reduction potentials of proton reduction, water oxidation and triethylamine oxidation (all potentials calculated at pH = 0, the numbers along the x-axis correspond to the number of phenylene moieties in the oligomer minus one, in line with the nomenclature used in the main paper).

References

(1) Aristizabal, J. A.; Soto, J. P.; Ballesteros, L.; Munoz, E.; Ahumada, J. C. *Polym Bull* **2013**, *70*, 35.

(2) (a) Hreha, R. D.; George, C. P.; Haldi, A.; Domercq, B.; Malagoli, M.; Barlow, S.; Bredas, J. L.; Kippelen, B.; Marder, S. R. *Adv. Funct. Mater.* **2003**, *13*, 967. (b) Sprick, R. S.; Hoyos, M; Morrison, J. J.; Grace, I. M.; Lambert, C.; Navarro, O.; Turner, M. L. *J. Mater. Chem. C* **2013**,*1*, 3327.

(3) Fung, B. M.; Khitrin, A. K.; Ermolaev, K. *J. Magn. Reson.* **2000**, *142*, 97.

(4) Morcombe, C. R.; Zilm, K. W. *J. Magn. Reson.* **2003**, *162*, 479.

(5) M. Jayakannan, J. L. J. van Dongen, R. A. J. Janssen, *Macromolecules* **2001**, *34*, 5386.

(6) Kolossváry, I.; Guida, W. C. *J. Am. Chem. Soc.* **1996**, *118*, 5011.

(7) Becke, A. D. *J. Chem. Phys.* **1993**, *98*, 5648.

(8) Lee, C.; Yang, W.; Parr, R. G. *Phys Rev B.* **1988**, *37*, 785.

(9) Vosko, S. H.; Wilk, L.; Nusair, M. *Can. J. Phys.* **1980**, *58*, 1200.

(10) Stephens, P. J.; Devlin, F. J.; Chabalowski, C. F.; Frisch, M. J. *J. Phys. Chem.* **1994**, *98*, 11623.

(11) Schafer, A.; Horn, H.; Ahlrichs, R. *J. Chem. Phys.* **1992**, *97*, 2571.

(12) Ahlrichs, R.; Bär, M.; Häser, M.; Horn, H.; Kölmel, C. *Chem. Phys. Lett.* **1989**, *162*, 165.

(13) Furche, F.; Ahlrichs, R. *J. Chem. Phys.* **2004**, *121*, 12772.

(14) van Wüllen, C. *J. Comput. Chem.* **2011**, *32*, 1195.

(15) Valiev, M.; Bylaska, E. J.; Govind, N.; Kowalski, K.; Straatsma, T. P.; Van Dam, H. J. J.; Wang, D.; Nieplocha, J.; Apra, E.; Windus, T. L.; de Jong, W. A. *Comput. Phys. Commun.* **2010**, *181*, 1477.

(16) Guiglion, P.; Butchosa, C.; Zwijnenburg, M. A. *J. Mater. Chem. A* **2014**, *2*, 11996.

(17) Butchosa, C.; Guiglion, P.; Zwijnenburg, M. A. *J. Phys. Chem. C* **2014**, *118*, 24833.

(18) Trasatti, S. *J Electroanal. Chem. Interfacial Electrochem.* **1986**, *209*, 417.
